# Optimizing expression quantitative trait locus mapping workflows for single-cell studies

**DOI:** 10.1186/s13059-021-02407-x

**Published:** 2021-06-24

**Authors:** Anna S. E. Cuomo, Giordano Alvari, Christina B. Azodi, single-cell eQTLGen consortium, Davis J. McCarthy, Marc Jan Bonder

**Affiliations:** 1 European Molecular Biology Laboratory, European Bioinformatics Institute (EMBL-EBI), Hinxton, Cambridge, UK; 2 Wellcome Trust Sanger Institute, Hinxton, Cambridge, UK; 3 Division of Computational Genomics and Systems Genetics, German Cancer Research Center (DKFZ), Heidelberg, Germany; 4 St. Vincent’s Institute of Medical Research, Fitzroy, Victoria Australia; 5 University of Melbourne, Parkville, Victoria Australia; 6 European Molecular Biology Laboratory, Genome Biology Unit, Heidelberg, Germany

## Abstract

**Background:**

Single-cell RNA sequencing (scRNA-seq) has enabled the unbiased, high-throughput quantification of gene expression specific to cell types and states. With the cost of scRNA-seq decreasing and techniques for sample multiplexing improving, population-scale scRNA-seq, and thus single-cell expression quantitative trait locus (sc-eQTL) mapping, is increasingly feasible. Mapping of sc-eQTL provides additional resolution to study the regulatory role of common genetic variants on gene expression across a plethora of cell types and states and promises to improve our understanding of genetic regulation across tissues in both health and disease.

**Results:**

While previously established methods for bulk eQTL mapping can, in principle, be applied to sc-eQTL mapping, there are a number of open questions about how best to process scRNA-seq data and adapt bulk methods to optimize sc-eQTL mapping. Here, we evaluate the role of different normalization and aggregation strategies, covariate adjustment techniques, and multiple testing correction methods to establish best practice guidelines. We use both real and simulated datasets across single-cell technologies to systematically assess the impact of these different statistical approaches.

**Conclusion:**

We provide recommendations for future single-cell eQTL studies that can yield up to twice as many eQTL discoveries as default approaches ported from bulk studies.

**Supplementary Information:**

The online version contains supplementary material available at 10.1186/s13059-021-02407-x.

## Introduction

Expression quantitative trait locus (eQTL, see Table 1) mapping is an established tool for identifying genetic variants that play a regulatory role in gene expression. The approach has been widely applied to bulk RNA sequencing profiles from primary human tissues [2] and cell lines [3, 4], as well as sorted cell populations, e.g., blood cell types [5]. Statistical methods for (bulk) eQTL mapping have been extensively tested over the years, with key findings including the need to control for population structure and covariates [6] and to account for multiple testing to control the false discovery rate [7]. Linear mixed models (LMMs) in particular have become a popular framework for genetic analyses of molecular traits, due to their flexibility and ability to robustly control for confounding factors.

**Table 1:** Abbreviations used in the manuscript

	Definition
**eQTL**	Expression quantitative trait locus/loci
**sc-eQTL**	Single-cell eQTL
**eGene**	Gene with at least one eQTL (at a given FDR threshold)
**LMM**	Linear mixed model
**Matched datasets**	(Bulk and single-cell) expression data from the same set of individuals with closely matched expression quantification.
**a-bulk**	All bulk; eQTL results obtained using bulk RNA-seq data from all donors (*n* = 526)
**m-bulk**	Matched bulk; eQTL results using bulk RNA-seq from matched donors (*n* = 87)
**SV**	(Single-cell) sampling variation
**FDR**	False discovery rate
**cFDR**	Conditional FDR
**FWER**	Family-wise error rate
**HVGs**	Highly variable genes
**TMM**	Trimmed mean of M-values; normalization method proposed in [1]

Recent technological advances have allowed molecular phenotypes, including gene expression, to be assayed at the level of single cells. In particular, single-cell RNA sequencing (scRNA-seq) is now an established technique and can be deployed at population scale, across many individuals, by exploiting multiplexed experimental designs and using appropriate demultiplexing tools [8–10]. The ability to identify cell types and cell states in an unbiased manner from scRNA-seq data from a single experiment can be used to define homogeneous cell populations, quantify expression levels within them, and then map eQTL in each of them separately. Consequently, studies where single-cell expression profiles (rather than bulk) are used to perform eQTL mapping have emerged recently [10–16]. In addition to deciphering the cellular context in which genetic variants influence gene expression, this single-cell level approach gives us the opportunity to study gene regulation in rare cell types and cell types relevant to a disease or process of interest. Taken together, these advantages of scRNA-seq for eQTL mapping promise to greatly improve our understanding of the genetic architecture of gene regulation across tissues, in both human disease and development [17].

As scRNA-seq in large sample sizes becomes feasible, it is important to establish “best practices” and benchmark approaches for the design and analysis of genetic studies using single-cell data. Current efforts in this space have primarily focused on the experimental design of single-cell eQTL (sc-eQTL, Table 1) studies, assessing trade-offs between sequencing depth, the number of donors, and the cell count per donor [18, 19]. Such studies conclude that statistical power can be improved on a fixed budget by performing lower-depth sequencing on more cells per sample or on more total samples.

**Fig. 1:**
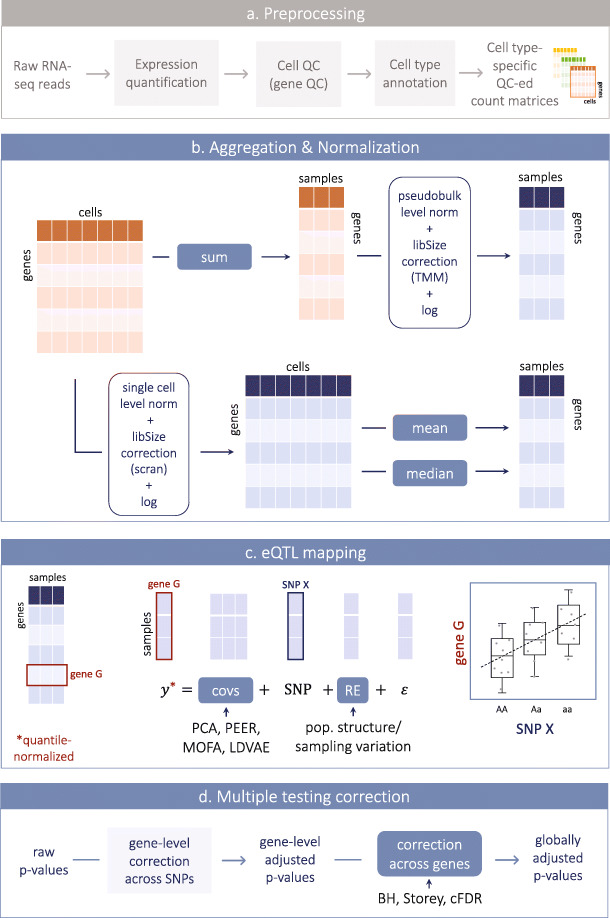
Overview of normalization, aggregation and the single-cell eQTL mapping considered. **a** RNA pre-processing steps to obtain count matrices to perform eQTL mapping, including gene expression quantification, cell and gene-level quality control (QC), and cell type annotation. These steps are not optimized/tested in this work (shown in gray). **b** Different approaches tested to perform eQTL mapping using scRNA-seq profiles. Starting from one gene x cell count matrix obtained as in **a**, counts were aggregated per sample (i.e., donor, or donor-run combination), either by summing the data first at the sample level and then normalizing using methods designed for bulk RNA-seq (i.e., TMM [1]) as implemented in edgeR or by first normalizing the single-cell counts (using scran/scater [20, 21]) and then calculating the mean or the median at the sample level. **c** eQTL mapping (cis). We map eQTL independently for each gene-SNP pair considered by fitting a linear mixed model. In particular, we model gene expression as the outcome variable (y), the SNP effect as well as additional covariates as fixed effects, and include one (or more) random effect (RE) term to account for population structure and sample variation. We considered various methods to compute covariates and tested different numbers of covariates as well. **d** Multiple testing correction is performed in two steps. First, gene-level p values are adjusted using a permutation scheme (“Methods”) to control the FWER across SNPs. Second, the top SNP per gene is selected (minimum adjusted p value; “Methods”) and various methods are used to control the FDR and obtain globally corrected p values. Steps that we optimize here are highlighted in blue in panels **b, c,** and **d**

However, the statistical methods needed to adapt bulk methods to map single-cell eQTL have not yet been systematically benchmarked. Notably, several important processing steps need to be performed on single-cell expression profiles before we can map the effects of genetic variants on them (Fig. 1a). First, cell-level gene expression counts can be obtained using a variety of different methods that are summarized and reviewed elsewhere [22–24]. Second, quality control (QC) steps should be performed at the level of single cells to filter out low-quality cells (see for example [25] for an overview of best practices). Additionally, individual sequencing runs and batches should be examined to determine their overall quality, and poor quality batches should be discarded altogether. Lastly, traditional genetic analysis of gene expression is well-defined within homogeneous cell populations. Thus, cell type assignment needs to be performed prior to genetic mapping. Clustering and cell type assignment algorithms and approaches have been widely described and remain a focus of benchmarking efforts [25, 26]. These processing steps are critical for successful downstream analyses like sc-eQTL mapping. However, because these processing steps are largely dataset- and/or technology-dependent and have been previously reviewed and benchmarked, here we focus on the steps after processing that are specific to eQTL mapping.

Specifically, we set out to optimize statistical analysis workflows that were originally developed for bulk RNA-seq studies in the sc-eQTL mapping setting. We take advantage of our unique, population-scale, dataset of matched (i.e., from the same individuals) single-cell and bulk expression data from a homogenous cell population (induced pluripotent cells; iPSCs [3, 27]) to evaluate the effect of different statistical choices. Because we have matched samples, we are able to measure performance both in terms of sc-eQTL yield (i.e., the number of significant sc-eQTL) and in terms of concordance with bulk eQTL, which can be considered as the current gold standard for eQTL mapping. We also validate these findings on simulated population-scale scRNA-seq datasets, where the eQTL effects are known, and performance can be measured in terms of power and false discovery rates. Using both empirical and simulated datasets, we identify best practices in terms of aggregation and normalization, the type and number of expression covariates to include as fixed effects, methods to account for single-cell sampling variation in the model, and methods to guide multiple testing correction using information from bulk RNA sequencing data. Finally, we demonstrate how applying these best practices increases power to detect sc-eQTL and reduce the number of false discoveries.

## Results

### Aggregation and normalization strategies

Traditional bulk eQTL are germline genetic variants that are associated with differences in gene expression between donors, where the gene expression values represent the summary of a gene’s expression across all cells in the tissue sample. In order to use traditional bulk eQTL mapping methods for single-cell eQTL mapping, we first need to aggregate the multiple measurements (i.e., cells in cluster X or cells of cell-type X) from each donor to obtain bulk-like measurements. Here, we explore different aggregation methods (Fig. 1b). In particular, we consider the mean, the median, and the sum as aggregation strategies. Initially, we performed aggregation at the donor (“d”) level, i.e., taking all cells for a donor, to maximize the numbers of cells per donor. We call the resulting methods “d-mean,” “d-median,” and “d-sum” (Table 2). To assess the impact of the method used to map eQTL, we compare four standard approaches, using correlation-based approaches (Pearson or Spearman), a linear model (LM) or a linear mixed model (LMM). We find that there is high concordance between eQTL identified using these methods and find that the correlation-based approaches have slightly lower replication rates as compared to the LM or LMM (Additional file 1: Table S1, Additional file 1: Fig. S1).

**Table 2:** Summary of the six key aggregation-normalization strategies used in this study. In particular, for each approach, we specify the aggregation method used, the type of normalization adopted, and the level of aggregation selected

	Definition		
	Aggregation method	Normalization	Aggregation level
**dr-mean**	Mean	Single-cell level (scran)	Donor and run
**dr-median**	Median	Single-cell level (scran)	Donor and run
**dr-sum**	Sum	Pseudo-bulk level (TMM)	Donor and run
**d-mean**	Mean	Single-cell level (scran)	Donor
**d-median**	Median	Single-cell level (scran)	Donor
**d-sum**	Sum	Pseudo-bulk level (TMM)	Donor

Given that single-cell RNA sequencing allows for pooling of multiple donors into single sequencing runs (i.e., batches), and for post hoc grouping of cells (for instance into cell types), we considered a second set of aggregation approaches, namely aggregating not only at the donor level but also for each individual sequencing run (i.e., all cells from a given donor in a single sequencing run within a single-cell type; designated “dr”, Table 2). We call the corresponding methods “dr-mean,” “dr-median,” and “dr-sum”. We chose to explore the dr aggregation level because in the datasets we considered some donors are present in multiple runs [13, 14]. In all cases (i.e., using any of the aggregation methods), aggregated expression values were only calculated for samples (i.e., donors or donor-run combinations) with at least 5 cells. This threshold was selected to be loose enough to minimize donor loss, while still eliminating donors with poor expression support. While the donor-run approach better accounts for variation across technical batches, it also introduces multiple measurements from the same donor. The LMM allows us to directly account for these repeated measurements, by including replicate and population structure information as random effect terms (“Methods”) and has higher replication rates than the simple regression and correlation-based methods, so we selected the LMM for our benchmark.

Importantly, normalization of the scRNA-seq data was performed in different ways depending on the aggregation method used. For the mean and median aggregation (both at the donor and the donor-run level), we performed single-cell-level normalization using scran [21] implemented in scater [20], which is one of the standard methods used for single-cell normalization. The mean and the median were then calculated on the resulting normalized (logged) counts (Fig. 1b, Tables 1 and 2). We also tested two other single-cell normalization approaches, bayNorm [28] and sctransform [29] (10X only), which produced correlated, but not identical, expression levels (Additional file 1: Table S2, “Methods”). For sum aggregation, summed count values (both dr-sum and d-sum) were obtained directly from the raw count data (i.e., non-normalized). Normalization was then applied on the resulting pseudo-bulk counts, using methods typically used for bulk RNA-seq data. In particular, we perform TMM normalization on the aggregated counts, as implemented in edgeR [30], one of the best-established methods for bulk RNA-seq normalization (Fig. 1b, Tables 1 and 2), followed by log transformation.

We tested these approaches on two empirical datasets and two simulated datasets. The main analyses were performed on expression data gathered from iPSCs, for which we have bulk RNA sequencing [27] and Smart-Seq2 single-cell RNA sequencing [13] data. This unique data allowed us to compare approaches to discovery of eQTL in single-cell expression data, using bulk eQTL results from the same samples as the gold standard for evaluating approaches for single-cell data. Specifically, we assessed the numbers of eGenes discovered in single-cell data and the extent of replication of bulk eQTL effects in single-cell analyses. Additionally, we considered one cell type (midbrain floor plate progenitor; FPP) from a large 10X single-cell RNA sequencing differentiation study, differentiating iPSCs towards dopaminergic neurons [14]. Lastly, to further corroborate and understand our findings, we tested our chosen approaches on simulated single-cell eQTL datasets generated using splatPop (“Methods”), based on either the Smart-Seq2 iPSC expression dataset or the 10X differentiating neuron dataset. In the simulations, we could assess eQTL discovery power and the fraction of false positives at a given FDR cutoff using the simulated eQTL effects.

### Mapping eQTL using aggregated Smart-Seq2 expression profiles

We first focused on the single-cell data from Cuomo et al. [13], from which we selected the iPSC data (day 0) to compare to bulk data from the HipSci consortium [3, 27]. We selected the iPSC data because the homogeneous expression profiles of these cells make it an ideal cell type for performing this kind of study, emulating the generic task of eQTL mapping for a single cell-type population from an scRNA-seq dataset. From the HipSci resource, we selected 87 healthy donors of European descent for which we had quality controlled: (1) genotype data, (2) Smart-Seq2 scRNA-seq, and (3) bulk RNA-seq (i.e., matched bulk, from here on “m-bulk”). Additionally, we used a superset of 526 samples (including the previous 87) for which we had quality controlled: (1) genotype data and (2) bulk RNA-seq data, for reference (i.e., all bulk, “a-bulk”). We (re)processed the raw RNA-seq data from the single-cell study to match the bulk processing as much as possible (“Methods”).

We aggregated the single-cell information as described above (Fig. 1b) and we tested for *cis*- expression quantitative trait loci (eQTL) using an LMM as implemented in LIMIX [31], considering SNPs within 100 kb around the gene body and with minor allele frequency (MAF) > 10% and Hardy-Weinberg equilibrium P < 0.001. We included in the model the first 20 expression principal components (PCs; based on the relevant aggregation method) and used an identical-by-descent kinship matrix to reflect population structure and replicated donors. Each gene’s expression was quantile-normalized prior to being included in the model as phenotype to better suit the assumptions underlying the LMM. We considered the set of 20,545 highly variable genes (HVGs) based on the single-cell measurements (“Methods”). In some instances, to facilitate comparison between the different aggregation/normalization strategies, we selected the set of common HVGs that were tested in every *cis*-eQTL map (n = 12,720 genes).

We identified between 776 and 1835 genes with at least one eQTL (from hereon: “eGenes,” FDR < 5%; “Methods”) using the different aggregation methods (out of 12,720 genes tested). To put these numbers in context, the equivalent eQTL map using matched samples with bulk RNA-seq identified 2590 eGenes (Table 3). The large difference in eGene discovery power between the bulk and single-cell methods is at least in part explained by the large difference in the total number of reads per donor and its variability across donors (Additional file 1: Fig. S2).

**Table 3:** Number of eGenes and replication of eQTL for the different aggregation and normalization strategies in Smart-Seq2 iPSC cells. The same set of 12,720 genes were considered in all of the strategies. Discovery FDR was controlled at 5% for the discovery; replication was defined as FDR < 10% and consistent direction of effect in the two bulk studies, i.e., matched bulk (*N* = 87, m-bulk) and all bulk set (*N* = 526, a-bulk)

	Discovery (FDR 5%)		m-bulk (FDR 10%)		a-bulk (FDR 10%)	
	eGenes	% tested	# replicated	% replicated	# replicated	% replicated
**dr-mean**	1835	14.43%	889	48.45%	1367	74.50%
**dr-median**	1337	10.51%	650	48.62%	952	71.20%
**dr-sum**	1463	11.50%	819	55.98%	1153	78.81%
**d-mean**	1305	10.26%	768	58.85%	1046	80.15%
**d-median**	776	6.10%	470	60.57%	625	80.54%
**d-sum**	1174	9.23%	709	60.39%	951	81.01%
**m-bulk**	2590	20.36%	–	–	2448	94.52%

Overall, we observe two main trends. First, aggregation at the donor-run level outperforms aggregation at the donor level only (dr-mean (1835) vs d-mean (1305); dr-median (1337) vs d-mean (776); dr-sum (1463) vs d-sum (1174)). Next, our results indicate that mean aggregation (after single-cell-specific normalization; 1835 eGenes) outperforms sum aggregation (followed by bulk-like normalization; 1463 eGenes), and median aggregation performs worst in all cases (1337 eGenes). As well as finding the lowest number of eGenes, the median methods are also responsible for the low intersection of HVGs from 20,545 to 12,720, due to cells with low read counts. When dropping median from the comparison the increase from d-mean to dr-mean is even higher (40% increase in eGenes considering all shared genes vs 46% increase in eGenes considering all tested genes, Additional file 1: Table S3). For consistency, we assessed sc-eQTL mapping on the bayNorm [28] dr-mean normalized data and found that the results were very similar compared to the other dr-mean results (1835 vs 1702 eGenes, and Pearson’s correlation between p values R = 0.95 and effect sizes R = 0.99, p value < 2.2 × 10^−16^ in both cases, Additional file 1: Table S4).

**Fig. 2:**
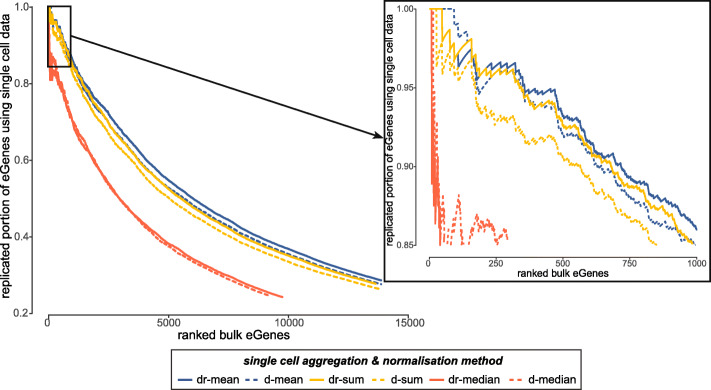
Replication of bulk eQTL in single cells. Replication rates of bulk eQTL in sc-eQTL as ranked by eGene significance (p value) in bulk. Shown are the replication rates of a-bulk eQTL for the six different aggregation/normalization approaches (mean, median, sum in blue, red, yellow respectively; dr aggregation level and d aggregation level in solid and dashed lines respectively)

Next, we used two selected sets of bulk iPSC RNA-seq data as described above, i.e., m-bulk (n = 87) and a-bulk (n = 526), to assess the replication of the iPSC sc-eQTL mapping results in bulk data (assumed to be the gold standard, Table 3, Fig. 2). We assessed replication of the top eQTL effects in a single-cell method in bulk (i.e., direct eQTL replication) and defined replication as FDR < 10% (in the replication set) and a consistent effect direction. Replication rates from the two sets of samples show a very similar picture: on average, we find slightly lower replication rates for the single-cell normalization methods, but a substantially higher total number of replicated discoveries at the eQTL level. In particular, the highest number of replicated eQTL is found for dr-mean (1450 considering a-bulk) and highest fraction of replication is found for d-sum (82%, a-bulk). Replication fractions remain consistent when considering all HVGs for eQTL (Additional file 1: Table S3). Moreover, we see higher replication rates considering a-bulk as compared to m-bulk, indicating that some of the effects found in the single-cell data can only be picked up from bulk datasets with more samples. When specifically looking at the effects that get replicated, we observe that they are highly overlapping (86%) across aggregation strategies. This result indicates that the same effects that get replicated in d-sum are also replicated in dr-mean, but since there are more effects found in dr-mean, the fraction is lower. In addition to assessing bulk replication, we used allele-specific expression (ASE) to directly validate eQTL effects by assessing allelic expression in the single-cell data. Replication rates of eQTL in ASE are lower 30–45%) but show the same trends in terms of replication of eQTL effects as the bulk eQTL replication (Additional file 1: Table S3).

Next, we set out to investigate which bulk eQTL could be identified using single-cell data to better understand what is driving eQTL identifiability in single-cell expression (Additional file 1: Fig. S3). We considered expression levels, expression variation, eQTL effect size (in the results using bulk expression), and genomic distance between eGene and eSNP. Unsurprisingly, we find that strong eQTL effects can be picked up using all methods, and eGenes with high expression and low variance are also easier for all methods to identify. Using the dr-median eQTL mapping approach, we fail to identify eQTL on lowly expressed genes, whereas dr-mean finds most eQTL effects. When investigating which single-cell eQTL could be replicated in the bulk results, we noted that this was mostly linked to the eQTL effect size, both in the single-cell and bulk eQTL results (Additional file 1: Fig. S4).

**Fig. 3:**
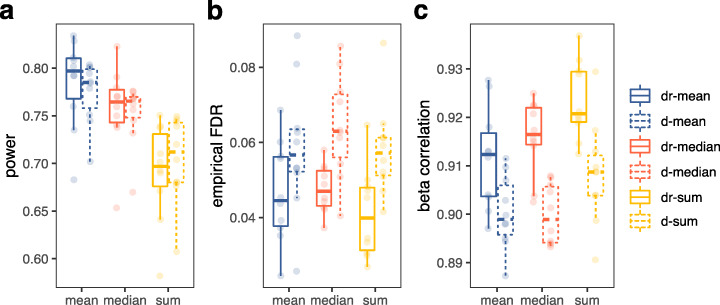
Summary of eQTL mapping performance on simulated Smart-Seq2 iPSC datasets. **a** Power to detect simulated eQTL (# true positives / # simulated eQTL). **b** Empirical FDR (false discovery rate at FDR < 5%, dashed line). **c** Pearson’s correlation between the ground truth and estimated effect sizes for genes simulated as eGenes. Colors and line types are as in Fig. 2. Box plots summarize the distribution, while the points show performance for each replicate (*n* = 10)

While the percentage of bulk eQTL replicated by sc-eQTL mapping in a matched dataset is a powerful performance metric, a limitation of empirical benchmarks like this is that the ground truth is not known. To validate our results on a dataset with completely known true eQTL effects, we simulated single-cell expression profiles for genes on chromosome 2 for a population with known eQTL effects applied to 35% of genes (total expressed genes on chr2: n = 1255, eQTL Genes = 439). To ensure the simulations reflected real data, expression statistics from the iPSC Smart-Seq2 dataset were used to estimate key parameters used in the simulations (Additional file 1: Fig. S5; see “Methods” for details). We then performed aggregation, normalization, and eQTL mapping as described for the empirical study on 10 replicate simulated datasets and quantified performance in terms of power (i.e., fraction of true eQTL detected), empirical FDR (i.e., fraction of false eQTL detected at an FDR < 5%), and effect size correlation (see “Methods” for details). Mean aggregation resulted in greater power of detection than median (paired t-test; p.adj = 1.9 × 10^−9^) and sum (p.adj = 7.3 × 10^-11^) aggregation, regardless of aggregation level (repeat measures two-way ANOVA; F(2,18) = 0.022, p = 0.97; Fig. 3a, results Additional file 2: Table S5; detailed statistical analysis Additional file 1: Table S6). The aggregation level had a significant effect on the empirical FDR (F(1,9) = 15.05, p = 0.004)), with donor-run-level aggregation resulting in fewer false positive eQTL (paired t-test: p = 0.036) (Fig. 3b). Finally, we assessed performance in terms of the correlation between the simulated ground truth eQTL effect sizes and the estimated effect sizes (Fig. 3c). Here the interaction between aggregation method and level was also not significant (F(2,18) = 0.154, p = 0.85), but both were significant on their own, with donor-run performing better than donor-level aggregation (paired t-test: p = 6.43 × 10^−4^) and mean performing better than median (p.adj = 0.041) and sum (p.adj = 7.5 × 10^−5^). These results corroborate findings from the empirical data analysis showing that mean aggregation after single-cell level normalization performs better than sum aggregation followed by bulk-normalization methods. Further, by comparing the mathematical properties of the eQTL correctly detected by each aggregation method, we can tell that median aggregation tended to miss eQTL for genes with high variance and low mean expression, while sum aggregation tended to miss eQTL with small effect sizes (Additional file 1: Fig. S6).

A remaining question was if the aggregation level and method would impact eQTL detection power the same way for different sized mapping populations and with differences in the number of cells per donor. We used the simulation framework to simulate and map eQTL for populations ranging from 50 to 600 donors and for populations with the average number of cells per donor ranging from 50 to 1000. From this analysis, we found that as the number of donors increased the differences in performance between the mean and sum aggregation decreased (Additional file 1: Fig. S7a). However, mean aggregation continues to improve as the number of cells per donor increases, while sum aggregation plateaus (Additional file 1: Fig. S7b). Further, while median aggregation improves with the number of cells per donor, it does not outperform mean.

### Mapping eQTL using aggregated 10X expression profiles

To check the generality of the results obtained from Smart-Seq2 data, we proceeded to assess the same aggregation/normalization strategies on data generated with the 10X Chromium technology [32]. Recent large-scale single-cell studies have predominantly used 10X because of the lower cost and higher cell throughput of the droplet-based scRNA-seq technology, as compared to plate-based technologies such as Smart-Seq2 [33]. Though cell throughput is higher, most often the number of reads quantified is lower for 10X studies, and reads are from the 3′ or 5′ end of transcripts (or both), but not from full-length transcripts as in Smart-Seq2. We used data from a recent differentiation study of iPSCs towards dopaminergic neurons [14]. This dataset was more complex than the iPSC Smart-Seq2 dataset described above, as it contained cells from multiple cell types. To map cell-type-specific sc-eQTL, we selected only cells annotated as midbrain floor plate progenitor (FPP) cells (n-donors = 174, “Methods”). Because we did not have matching bulk RNA-seq data, we used eQTL results on brain tissues from the GTEx consortium as a proxy (“Methods”) to assess replication. Additionally, we quantified ASE on the single-cell expression data directly.

In general, we observed very similar effects as found in the Smart-Seq2 dataset (Table 4). When comparing across the normalization methods (scran, baynorm, and sctransform), we observe high concordance in eQTL discovery (1496 vs 1152 vs 1444 eGenes respectively, and Pearson’s correlation between p values and effect sizes R > 0.92, p value < 2.2 × 10^−16^ in all cases, Additional file 1: Table S2, Additional file 1: Table S3). When comparing the aggregation methods, again the donor-run methods (dr-mean, dr-sum and dr-median) outperformed donor methods (d-mean, d-sum and d-median), and as for Smart-Seq2, we observe that the single-cell normalization method with mean aggregation outperformed the sum-based aggregation followed by bulk-like normalization, supporting the previous observation (both from the simulated and the empirical Smart-Seq2 iPSC data) that single-cell-specific normalization and treating different runs as separate entities is beneficial. Moreover, for 10X data, single-cell normalization with median aggregation performed especially poorly, likely due to the greater sparsity in 10X data compared to Smart-Seq2 data. Again, we observe that when considering all HVGs we see a larger increase of eGene discoveries from d-mean to dr-mean (19% increase compared to the initial 16%; Additional file 1: Table S7). We assessed replication of the FPP eQTL using bulk eQTL summary statistics from the GTEx mature brain tissues. Importantly, FPP cells are not mature brain cells, so these comparisons are useful but not perfect. We find again that the replication fraction of the donor results is higher as compared to donor-run eQTL, but the total number of replicated effects is higher for the donor-run-based methods (Additional file 1: Table S7). This picture is also reflected in the ASE replication, though the replication of eQTL in ASE is especially low for the 10X dataset (Additional file 1: Table S7).

**Table 4:** Number of eGenes for the different aggregation and normalization strategies in 10X midbrain floor plate progenitor cells. In total, 3504 genes were considered in all of the strategies, and gene-level FDR was controlled at 5%

	eGenes	% genes tested
dr-mean	1496	42.69%
dr-median	918	26.20%
dr-sum	1041	29.71%
d-mean	1252	35.73%
d-median	575	16.41%
d-sum	703	20.06%

To get a better idea on the power and replication of 10X sc-eQTL, we again validated these results using simulated data based on expression statistics from the neuron differentiation 10X dataset to estimate key parameters used in the simulations (see “Methods” for details). The simulations confirmed that median aggregation performs very poorly on 10X data (Additional file 1: Fig. S10). However, there was no significant difference between mean and sum aggregation or between donor and donor-run aggregation on power or empirical FDR (see results Additional file 2: Table S5, see detailed statistical analysis Additional file 1: Table S8). These patterns were generally consistent as the number of donors (Additional file 1: Fig. S11a) and the average number of cell-per-donor changed, with a notable exception that sum slightly outperformed mean aggregation with large numbers of cells per donor (Additional file 1: Fig. S11b).

### Mapping eQTL using individual cell expression profiles

As an alternative to the aggregation-based approaches, we also considered treating individual cells as distinct observations and mapping eQTL directly on single cells (after scran normalization, with no aggregation). An LMM is often used for “repeated measures” analyses—where multiple measurements are made from the same subject—in preference to classical approaches such as repeated measures ANOVA [34]. Thus, we tested an LMM for direct single-cell eQTL mapping by including a random effect term to account for the expected correlation between cells from the same donor as well as genetic relatedness between donors (“Methods”). We tested this approach on the subset of HVGs on chromosome 2 and considered two distinct approaches: first, we considered all available cells (ranging from 5 to 379 cells per individuals, *n* = 7552 cells, 2766 genes tested); second, we subsampled to a fixed number of cells (5 cells for each individual, n = 445, 2718 genes tested) per individual. In both cases, we observed a high number of eGenes (1668 for the first approach, corresponding to ~ 60% genes tested and 840 using the second approach, i.e., 31% of the genes tested). However, the replication rates were very low, ranging from 10 to 15% when considering the two approaches and the two sets of bulk results (“Methods”), suggesting inflation and a high rate of false positives, confirmed by poor correlation of effect sizes between these results and those obtained using bulk RNA-seq (Additional file 1: Fig. S8).

To further confirm this result, we tested direct single-cell eQTL mapping on one of the simulated datasets. Compared to dr-mean aggregation, using all available cells or 10 cells per donor reduced eQTL detection power by 56% and 67% and increased the empirical FDR from 0.07 to 0.68 and 0.36, respectively (Additional file 1: Fig. S9a), with little change in the correlation between estimated and simulated effect sizes for significant eQTL (Additional file 1: Fig. S9b). Direct single-cell eQTL mapping also substantially increased the computational burden, especially when including all available cells (Additional file 1: Fig. S9a). Finally, we tested SCeQTL [35], a bespoke single-cell eQTL mapping model that uses a zero-inflated negative binomial regression. While the considerable computational time required for SCeQTL precluded us from applying it to all available cells, on 10 cells per donor SCeQTL had 64% less power than dr-mean and an empirical FDR of 0.93, worse than the comparable LMM. Similar to the aggregation approaches, the single-cell mapping approaches are best able to capture true eQTL associations with large effect sizes and where the eGene has low variance, high expression, and fewer zeros (Additional file 1: Fig. S9c). Given single-cell LMMs and SCeQTL models do not perform better on a different type of eQTL, their overall higher false positive rate, and their increased computational burden, we focus on the aggregation-based approaches for the remainder of this paper.

### Correcting for global expression covariates

Another important step when mapping eQTL is the correction for batch effects and other known and hidden sources of unwanted variation in expression data [2, 36, 37], hereafter collectively referred to as covariates. In the previous analyses, we used the first 20 PCs as covariates, which is common practice in eQTL studies [36, 37]. Here, we tested the impact of alternative approaches and different numbers of factors to include as fixed effect covariates in the LMMs used for sc-eQTL mapping. We compared multiple different methods to capture global expression covariates: probabilistic estimation of expression residuals (PEER [38]), principal component analysis (PCA), linearly decoded variational autoencoder (LDVAE or linear scVI [39]), and multi-omic factor analysis (MOFA [40]), for which we considered two different flavors: with and without sparsity constraints; “Methods”). For each approach, we tested the effect of including 5–25 factors as covariates in the LMM, in steps of five. We again considered the iPSC data as a homogeneous cell type for which we have both bulk RNA-seq and single-cell RNA-seq on the same samples available. For these tests, we focus on the dr-mean aggregation method, as it performed best in both the simulations and empirical tests.

**Fig. 4:**
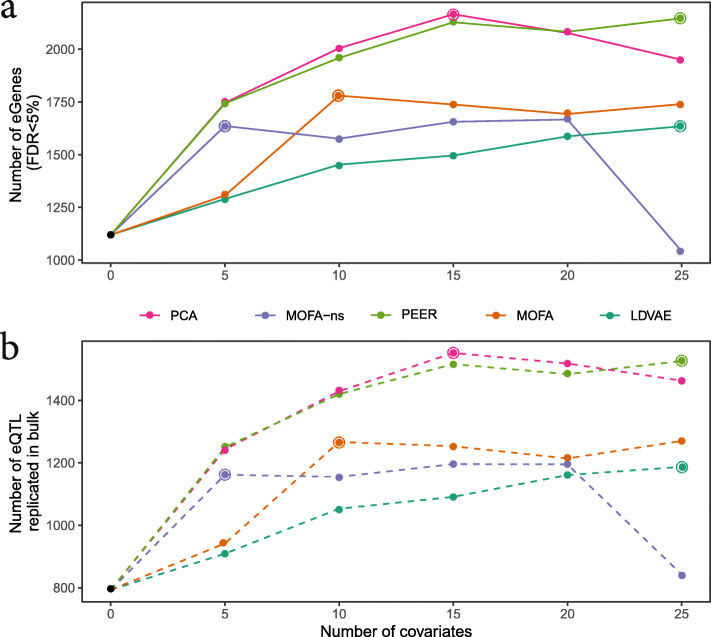
Comparison between covariate adjustment approaches. **a** Number of eGenes obtained when using different approaches to account for covariates, as a function of the number of factors used as covariates (out of 20,545 genes tested)**. b** Number of replicated discoveries using a-bulk (all samples, n = 526). Replication defined as FDR < 10% and consistent direction of effect. The optimal number of covariates for each method is highlighted with an open circle

As previously described, we observed a big increase in the number of eGenes discovered when considering covariates as compared to not considering covariates: the minimum increase is 75% (Fig. 4) [38]. However, when comparing the method-specific optimal number of covariates (e.g., 15 for PCA, 25 for PEER, Fig. 4, “Methods”), we observe that both PCA and PEER perform markedly better than the other methods (Additional file 1: Table S9, Fig. 4a). Both the sparse and non-sparse modes of MOFA perform worse and LDVAE, the only method included that works directly on the single-cell data produces the smallest increase in eGene discovery. Furthermore, the replication rates of the effects in a-bulk, fixed at 20 PCs as previously used, are similar between the different methods (Fig. 4b). Our results also show that more computationally expensive methods such as LDVAE, PEER, or MOFA do not perform measurably better than the historical default in bulk eQTL studies of correcting for unwanted variation using principal components.

### Accounting for single-cell sampling variation

After optimizing the aggregation-normalization method and the covariate correction, we moved on to test the effect of accounting for “sampling variation” (SV) in single-cell expression quantifications by including a (second) random effect in our linear mixed model that captures sampling effects (similar to the approach used in [14]; “Methods”). We hypothesized that the total number of reads used to quantify expression per sample and the number of QC-passing cells per sample could contribute to single-cell SV, so we tested the inclusion of random effects defined by these features in the LMM. First, we focused on the d-mean results and replaced the random effect accounting for kinship (baseline) with random effects accounting for either 1/#reads or 1/#cells. Second, we considered dr-mean, which had higher power in eQTL mapping, and tested the use of a joint random effect accounting for both kinship and SV, again either 1/#reads or 1/#cells (in this case, we cannot remove the kinship effect term, which reflects the replicated structure of samples across batches, see “Methods” for details).

For d-mean, we observe that replacing the kinship-based random effect with the random effects reflecting SV increases *cis*-eQTL mapping power by > 9% (Table 5). Additionally, replication rates are similar across all tests, indicating that the additional effects are as likely to be true effects as the initially discovered eQTL. When considering the dr-mean results, we observe an even stronger increase in eGene discovery power (> 21%) when accounting for SV alongside the replicate structure. Again, we observe that the replication rates are comparable to the baseline results (kinship/replicate structure only). For both the d-mean and dr-mean results the 1/#cells random effect seems to work better than 1/#reads.

**Table 5:** Inclusion of random effect to increase discovery power of sc-eQTL mapping in Smart-Seq2 iPSC scRNA-seq data. Shown are the number of eGenes that are discovered at an FDR of 5% and the replication in all bulk (a-bulk) defined as FDR < 10% and same sign. Tested are the expressed genes on chromosome 2 matched between the two considered aggregations (d-mean and dr-mean, n genes = 20,334)

	Random effect matrix			Discovery		a-bulk replication	
	kinship	1/#cells	1/#reads	eGenes	% tested	# replicated	% replicated
**d-mean**	✓	–	–	1415	6.96%	1086	76.75%
**d-mean**	–	✓	–	1652	8.12%	1251	75.73%
**d-mean**	–	–	✓	1645	8.09%	1233	74.95%
**dr-mean**	✓	–	–	2073	10.19%	1466	70.72%
**dr-mean**	✓	✓	–	2750	13.52%	1832	66.62%
**dr-mean**	✓	–	✓	2458	12.09%	1676	68.19%

Next, we assessed the impact of the inclusion of the SV measure on eQTL mapping in the 10X dataset. Given the lower read-depths per cell in 10X versus Smart-Seq2, we expected a stronger effect in this setting. However, for the d-mean results, we observe a slightly smaller (~ 4%) increase in eGene discovery than was observed in the iPSC Smart-Seq2 results (Additional file 1: Table S10). For the dr-mean based methods, where we include both kinship and SV, we observe again a stronger increase in eGene discovery of around 20%. Like for Smart-Seq2, the SV effect based on 1/#cells allows for the identification of more eGenes than the 1/#reads-based random effect.

To get further insight into the effects of the (additional) random effect accounting for sampling variation on the power to detect sc-eQTL, we added the SV term when mapping eQTL on the simulated scRNA-seq data. For both Smart-Seq2 and 10X simulations, adding either 1/#cells or 1/#reads as an additional random effect resulted in an increase in the number of true eQTL detected (Additional file 1: Table S11). However, it also tended to increase the number of false discoveries. We would expect the effect of sampling variation to be the largest when there are large disparities between the number of cells or number of reads between observations, and thus including sampling variation in the model to be most useful in these cases. This conclusion is supported by our finding that the addition of the SV random effect resulted in the largest gain and lowest loss in the case of donor-level aggregation on the 10X data.

### Guided multiple testing increases discovery power

**Fig. 5:**
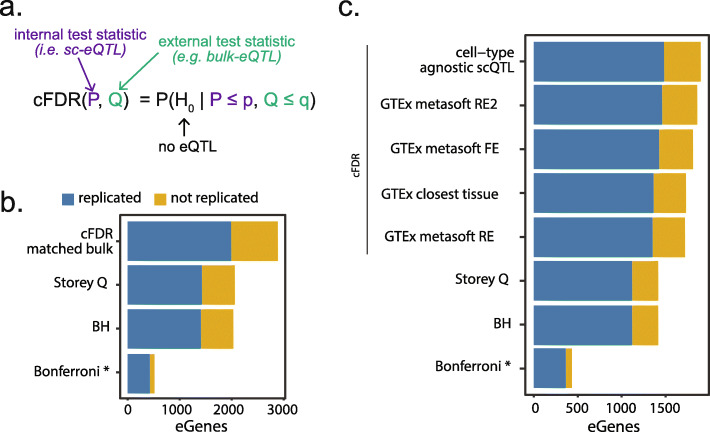
Conditional FDR increases eGene discovery in iPSC Smart-Seq2 data while replication fraction stays consistent. **a** Graphical summary of cFDR method. **b** The eGene discovery power using different FDR methods at an FDR of 5% for the respective method (p < 0.05 Bonferroni). Blue bars indicate eGenes that were replicated in all bulk (a-bulk) defined as FDR < 10% and the same direction of effect. Yellow bars indicate eGenes that were not replicated in bulk (number of total genes tested = 20,334). **c** The eGene discovery power using different external test statistics for cFDR. Tests were performed only on the subset of genes present in all four external datasets (GTEx metasoft RE2, FE, RE, and closest tissue) (13,653 genes tested). * denotes multiple testing correction methods that control the (much stricter) family-wise error rate

Given that the discovery power of eQTL is heavily dependent on the number of donors for which genetic and expression data is available, and large bulk eQTL studies are available, we set out to leverage bulk data to increase discovery power when mapping sc-*cis*-eQTL. To this end, we selected a recently proposed multiple testing correction method that conditions the false discovery rate (i.e., conditional FDR, or cFDR) on an external set of test statistics [41, 42] and tested the impact of its application to sc-eQTL mapping (Fig. 5a).

To assess the impact of using cFDR, we used the dr-mean iPSC results and the eQTL statistics of the m-bulk samples to condition our FDR estimates on and used a-bulk samples to test the replication rates (Fig. 5b, Additional file 1: Table S12). The cFDR eQTL results are compared against three “standard” multiple testing correction methods in eQTL mapping (Bonferroni [43], Benjamin-Hochberg [44], and Storey Q [45]—the latter being the standard method for our analyses [27, 46–48]). We observed a 40% increase in eGene discovery by applying cFDR as compared to Storey Q, the second-best performing method in terms of eGenes. When assessing the replication rates in a-bulk of the eQTL effects deemed significant by the different multiple testing procedures, we observe very similar replication rates for the methods seeking to control the false discovery rate. The Bonferroni method seeks to control the (much stricter) family-wise error rate, and as such has a higher replication rate but at the cost of making many fewer discoveries. Importantly, all eQTL identified by Storey Q are also significant in cFDR. In this specific case, we could apply cFDR when only changing the expression data but keeping other settings matched, i.e., same donors and therefore the same genotypes. Because it is rare to have matched bulk and scRNA-seq data for the same individuals, this is not a realistic setting for most applications of cFDR for sc-eQTL mapping.

To generalize this approach, we applied cFDR using other reference eQTL statistics to guide our sc-eQTL mapping analysis. Specifically, we selected two external reference sets from GTEx: (1) the lymphocyte eQTL summary statistics from GTEx (v7) and (2) the meta-tissue eQTL results by GTEx (v7). For the meta-tissue results, we assessed all different meta-analysis p values (i.e., the fixed effect, random effect, and random effect 2 as calculated using metasoft [48]). Lastly, we used a cell-type agnostic eQTL mapping on the two other differentiation time points presented in the Cuomo et al. study (i.e., mesendoderm and definitive endoderm). This final setting should be useful in most common sc-eQTL mapping settings where either a cell-type-specific or cell-type agnostic analysis can be performed within the same study. Similar to the results from the matched-sample cFDR, we observe that all of the cFDR methods (whichever test statistics are used for conditioning) outperform the Storey Q-based multiple testing correction in terms of number of eGenes discovered (Fig. 5c, Additional file 1: Table S13). Moreover, replication fractions are highly similar between the different FDR approaches (Storey Q and BH vs cFDR). The use of the p values from the same study provided the largest increase in the number of eGenes, but the use of the metasoft p values from the GTEx tissues is a close second (1898 vs 1859 eGenes). Of importance here is that for Additional file 1: Table S13 we subsetted down to only consider genes tested in all datasets, to be able to compare between cFDR conditionings. When assessing the impact per reference p value set, we see that leveraging the joint cell types from the Cuomo et al. study yields a comparable number of discoveries as using the m-bulk data (2821 vs 2887 eGenes, with comparable replication fractions), and observe that the GTEx summary statistics do considerably worse as the number of overlapping expressed genes is much lower (Additional file 1: Table S14).

### Optimized sc-eQTL mapping

The previous sections describe how to optimize individual steps during a sc-eQTL mapping workflow, including steps specific to single-cell aggregation and normalization, statistical modeling, and multiple testing correction. Here we describe our combined optimized sc-eQTL mapping workflow and showcase the total increase in power possible when combining the individual findings. We directly compared the eGene discovery results from a default workflow using d-mean aggregation (baseline) to dr-mean with an additional random effect to capture sampling variation and an improved multiple testing strategy applied. Again, we focused here on the iPSC data to be able to assess replication in bulk.

**Fig. 6:**
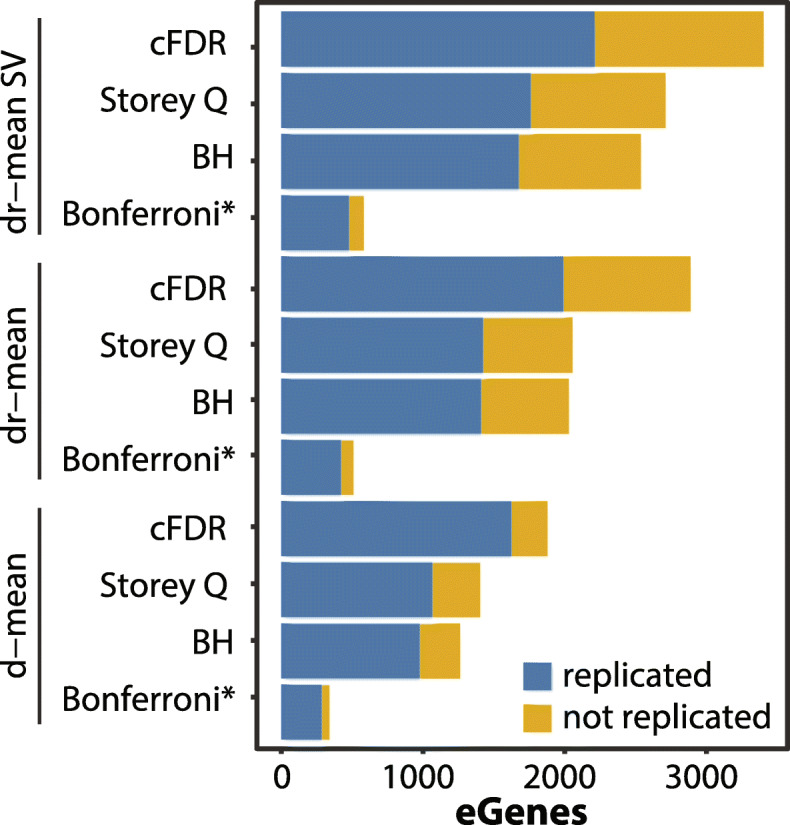
Optimizing the eQTL mapping workflow increases eGene discovery. The eGene discovery power after optimizing for aggregation level, including random effect reflecting sampling variation (SV, 1/#cells), and applying cFDR methods (conditioning on matched bulk p values) at an FDR of 5% for the respective method (p < 0.05 Bonferroni). Blue bars indicate eGenes that were replicated in all bulk (a-bulk) defined as FDR < 10% and the same sign. Yellow bars indicate eGenes that were not replicated in bulk (genes tested = 20,334). * denotes multiple testing correction methods that control the (much stricter) family-wise error rate

Overall, we observe a 142.7% increase in eGenes detected when comparing the d-mean aggregation with Storey Q-based multiple testing correction (1402 eGenes) versus the optimized eQTL mapping approach, specifically dr-mean with additional random effect and cFDR multiple testing (3402 eGenes, Fig. 6, Additional file 1: Table S15). This drastic increase is explained by the smaller individual optimizations discussed above. The switch to dr-mean instead of d-mean increases mapping power by 46.6%, the use of the additional random effect reflecting SV (1/#cells) increases power by an additional 31.9%, and lastly the guided multiple testing correction using p values from the matched bulk analysis increases power by 25.5%. In terms of how well these sc-eGenes replicated the bulk-eGenes (using FDR < 10% and same effect direction as replication criteria), we observe decreases in the fraction of sc-eGenes that replicated bulk-eGenes at each of the optimization steps: ~ 7% replication decrease for d-mean to dr-mean and an additional ~ 4% replication decrease for the additional random effect on sample variation. However, the total number of replicated bulk eQTL effects increased at every step, with 1146 (+ 107.4%) additional eQTL replicated with the optimized mapping compared to baseline. When using a more stringent multiple testing cutoff for eQTL discovery (FDR < 1%) in the optimal mapping procedure we observed a replication rate of 75.5%, which is similar to the replication rate for d-mean eQTL effects with Storey Q multiple testing (i.e., baseline), while still identifying 548 (51%) additional eGenes (Additional file 1: Table S16).

## Discussion

Since being highlighted as “Method of the Year” in 2013 [49], sequencing of the genetic material of single cells has become common practice to investigate cell-to-cell heterogeneity in biological systems [50]. Now an established method, scRNA-seq is being applied on larger and larger scales, for example to chart all the cell types in the human body (Human Cell Atlas (HCA, [51])) and to study the differences between individuals from population-scale cohorts. This latter development has enabled us to study the effects of genetic variation on gene expression, at single-cell resolution. Recent studies have explored the optimization of eQTL discovery power as a function of budgetary constraints; however, limited attention has been paid so far to optimizing statistical workflows for eQTL mapping to improve power for the mapping of eQTL with the selected design.

Here, we set out to assess the effects of different eQTL mapping workflows for single-cell studies. We used bulk and single-cell expression profiles derived from the same iPSC lines to assess both power and replication of genetic effects on gene expression variation. We also tested both Smart-Seq2 and 10X data to determine how our recommendations would hold across technologies. To support our empirical data, we used splatPop to simulate data with known eQTL effects.

We find that optimizing the aggregation and normalization approaches can increase eQTL mapping power substantially: in the Smart-Seq2 study, we identify close to two times more eGenes for the best performing method vs a non-optimized baseline method. In the 10X study, we find that the dr-mean approach also gives most power to identify eGenes, but we could not assess the replication in bulk. However, the 10X simulations show that dr-level aggregation results in fewer false discoveries and that both mean and sum aggregation perform well. We speculate that while the sum would perhaps be the most obvious approach to reproduce bulk-like measurements, the mean might perform better because of the cell-level normalization used. Indeed, the normalization at the cell level may better balance the differences in read counts across cells prior to the aggregation at the individual level. Additionally, it is of note that methods in which we treat replicates as separate samples (i.e., donor run) work best even though only 20 (23%) of the Smart-Seq2 study samples and 25 (14%) for the 10X study samples were assayed in more than one run. We expect that these improvements are also down to improved normalization as well as improved covariate correction. Finally, we tested median aggregation, but given the sparsity of the single-cell data this method drastically decreases the number of genes that can be assessed for eQTL mapping. Therefore, the use of median aggregation is not a sensible method in most single-cell applications.

Next, we assessed the effects of both covariate and sampling variation correction approaches. First, the effect of the different methods to identify covariates, i.e., data driven factors that capture unwanted variation in eQTL mapping, is ambiguous. Adding covariate factors of any method increases eGene discovery power substantially, but the results for the different tools when using the optimal number of factors for that tool are broadly comparable both in terms of discovery and number of replicated effects. Correcting for 15 PCs yielded the most eGenes at a high replication rate. Importantly, the bulk eQTL results we use to assess replication were mapped after correcting the bulk RNA-seq data using 20 PCs, which might bias the replication for the sc-eQTL mapping to be more similar to the bulk PCA-based results. However, regardless of the replication rate in bulk, PCA correction yielded the highest number of eGenes and so remains a sensible “default” choice for defining covariates capturing unwanted variation. Second, including sampling variation as a random effect in the models results in an increase in eGene discovery power on both Smart-Seq2 and 10X datasets, especially when using the 1/#cell-based random effect.

Finally, we explored the use of bulk eQTL mapping statistics to guide our multiple testing correction using the conditional false discovery rate (cFDR) in the implementation by Liley et al. We observe that the cFDR method gives a marked increase in the identified eGenes (between 21% and 40% increase), both when matched bulk data is available and when meta-tissue eQTL summary statistics are used. However, using the larger iPSC dataset, we found cFDR had limited impact in terms of replication rates of the eQTL. While cFDR allows us to guide the multiple testing, potential cell type-specific effects that are borderline significant might not be “boosted” in power by cFDR if those cell types are not present or lowly represented in the bulk references. We did, however, observe that all the effects found using standard FDR methods (Storey Q, Benjamini-Hochberg) were also deemed significant using cFDR, so no potential effects were lost by using the guided FDR approach. We explored the use of cFDR for other single-cell studies, but more tests are needed for mixed cell types to find the optimal way to use cFDR in sc-eQTL studies.

This study demonstrates that optimizing the sc-eQTL mapping workflow can increase eGene discovery power substantially. However, our conclusions come with some caveats; (1) simply discovering more eGenes does not necessarily mean that an approach is better, as false positives could arise due to data processing decisions; (2) bulk eQTL are a powerful, but not perfect, gold standard for assessing truth, as biases in bulk eQTL mapping may be replicated in sc-eQTL mapping analyses; (3) while our simulation framework uses parameters estimated from empirical data to resemble real population-scale scRNA-seq data, all simulation frameworks have limitations; (4) single-cell processing steps such as expression quantification, cell-level QC, and cell-type annotation could affect eQTL mapping performance, but best practices for these steps are likely to be dataset and technology specific rather than specific for sc-eQTL mapping, and were thus not assessed here. Also, the methods we explored here are based on current standards for bulk eQTL mapping. Though we find that directly using single-cell expression information does not yield trustworthy sc-eQTL, there is room for methodological improvements to better use the replicated structure of single-cell expression data and low counts in each cell.

In this paper, we discussed the impact of various normalization, aggregation, and covariate correction procedures as well as multiple testing correction in the context of single-cell eQTL mapping power. We conclude that following methodological choices are currently optimal: scran normalization; mean aggregation of expression across cells from one donor (and sequencing run/batch if relevant); including principal components as covariates in the LMM; including a random effect capturing sampling variation in the LMM; and accounting for multiple testing by using the conditional false discovery rate. Together our optimized sc-eQTL mapping workflow increases eGene discovery by more than a factor two in our empirical data. These findings should guide the community as more population-scale scRNA sequencing data becomes available and as groups like the single-cell eQTLGen consortium are establishing sc-QTL studies on a massive scale.

## Methods

### iPSC RNA-seq data

To optimize processing and workflows for eQTL mapping in single cells we focus on iPSC cells, as iPSCs have a homogeneous and stable expression profile [27] and because of the availability of both single-cell [13] and bulk expression [3, 27] data for iPSCs for the same donors through the HipSci consortium. Genotyping of the iPSC lines is described in the original study [3]. Expression quantification per technology is described below.

### Single-cell RNA-seq data processing

The Cuomo et al. [13] study describes in detail the differentiation of iPSCs towards definitive endoderm using a 3-day differentiation protocol. Briefly, the iPSC lines were multiplexed into 24 experimental pools (4–6 lines in each pool) and single cells were sequenced using the Smart-Seq2 protocol [33]). Cells were assigned to their donor of origin using Cardelino [8]. Here, we focus on the day 0 cells (i.e., iPSC stage) that were successfully assigned to a donor and passed donor- and cell-level QC described below.

First, we quantified gene expression levels in line with the bulk study. We mapped RNA-seq reads to the genome (hg19) using STAR [52] (version: 020201) mapping and used ENSEMBL v75 for annotation. Expression levels for each gene were counted using featureCount (subread v1.6.0 [53]). To remove outlier runs (i.e., pools), we (1) calculated the median correlation of each cell with all other cells (across all runs), then (2) for each run calculated the median of the resulting median cell-cell correlations, and finally (3) discarded cells with a median cell-cell correlation < 0.7, leaving 30 runs (out of 35, Additional file 1: Fig. S12a). Then, we recalculated the cell-cell correlations between cells from the remaining runs and discarded cells with a median cell-cell correlation < 0.5, leaving 7611 cells (corresponding to 155 donor-run combinations; Additional file 1: Fig. S12b). The cell QC applied here removed a sub-population of lower quality cells that would have been picked up by more traditional QC metrics, such as the total number of genes detected per cell (Additional file 1: Fig. S13).

Additionally, to remove further possible confounding effects, a small group of donors with monogenic diabetes (n = 10) and four donors that were outliers in the genotype space (based on projection with the 1000G project data) were excluded.

After QC and selection, the number of cells per donor varied from 5 to 383 iPSCs per donor (mean = 84.8) and the number of reads per donor varied from 1 to 104 million (mean = 42 million). For reference, the average number of reads per donor in our bulk data is 44 million, but the range is much smaller (24–98 million reads per person, Additional file 1: Fig. S2).

The two other developmental stages from Cuomo et al., i.e., mesendoderm and definitive endoderm, are used as a reference set for the cFDR analysis. Cell QC and cell assignment to these two developmental stages are described in the original publication [48]. However, gene expression quantification was performed in line with the single-cell iPSC data described above.

### Bulk RNA-seq data processing

The HipSci project generated both chip-genotype and deep bulk RNA-sequencing profiles for 810 iPSC lines corresponding to 527 unique donors. Details on expression quantification and QC of these data can be found in Bonder et al [27]. Of the 111 pre-QC donors for which we had single-cell RNA-seq data, matching bulk iPSC data was available for 108 (97%). After QC and selection on both the bulk data and the single-cell expression data, we were left with data from 87 donors.

### 10X data from floor plate progenitor cells

Recent large-scale single-cell expression studies have mostly been conducted using the 10X Chromium platform [32], given its larger throughput in terms of number of cells and lower costs per cell. The quantification of expression from 10X is different from Smart-Seq2, because 10X sequencing (1) only aims to capture sequence from the 3′, 5′, or both 3′ and 5′ ends of transcripts and (2) uses unique molecular identifiers to identify and remove effects of PCR duplication, and (3) results in fewer reads per cell compared to Smart-Seq2.

Given these differences and the recent shift towards 10X data, we wanted to assess the impact of eQTL workflows on 10X data specifically. We selected one cell-type (midbrain floor plate progenitors; FPP) and time-point (day 11 of iPSC differentiation towards dopaminergic neurons) from Jerber et al [14], using expression quantification from the original paper and the two-step cell QC as described above (as in Additional file 1: Fig. S12). Briefly, sequencing data generated from Chromium 10X Genomics libraries were processed using the CellRanger software (version 2.1.0) and aligned to the GRCh37/hg19 reference genome. Counts were quantified using the CellRanger “count” command, with the Ensembl 84 reference transcriptome (32,738 genes) with default QC parameters. For each of 13 pooled experiments, donors (i.e., cell lines) were demultiplexed using demuxlet [10], using genotypes of common (MAF > 1%) exonic variants available from the HipSci bank and a doublet prior of 0.05.

Sufficient single-cell data (≥ 5 cells) were available for 174 individual cell lines from 174 donors. The number of cells per donor ranged from 7 to 7782 (mean = 831.4). The total number of reads per donor ranged from 0.02 to 114 million reads (mean = 9.7).

### GTEx brain eQTL summary statistics

To assess the replication of 10X FFP eQTLs, we leveraged the brain summary statistics available from the GTEx study (v7) [2]. Given that there was no perfect bulk reference for the FPP cells and power is limited in the GTEx brain tissues, we first did a weighted average over the effect sizes in the different brain tissues. After deriving the effect sizes, we looked up the most significant SNP-level corrected p value [46] from the individual tissue associations to reflect this averaged effect size in the brain. When doing the lookup for the FPP cells, we additionally corrected these p values for the number of genes that are assessed in the relevant test.

### Allele-specific expression quantification and testing

In addition to bulk eQTLs results, we assessed allele-specific expression (ASE) levels as a second method of replication. We leveraged the WASP (version: 0.3.4) pipeline to quantify ASE on both the single-cell datasets (i.e., Smart-Seq2 and 10X) [54]. First, we merged the alignments at a donor level; second, we processed the imputed genotype data and the merged BAMs as outlined in the WASP instructions (https://github.com/bmvdgeijn/WASP/tree/master/CHT). To replicate the eQTL results, we used the WASP ASE counter to count genic reads overlapping phased variants linked to the eQTL SNP. For the gene counting, we use a processed gene annotation file that contains merged overlapping exons from the same gene and removed regions that are present in multiple genes as recommended by WASP. After counting, we used the bias corrections implemented in WASP to correct the read counts and used WASP to quantify ASE. Importantly, WASP was used as provided, meaning that no specific optimizations for single-cell ASE counting were used. Given the lower read counts in single-cell data, we could not replicate many eQTL results; however, the allelic expression ratios overlap the eQTL signs to a large extent. To correct for multiple testing in the ASE analysis, we used the Storey Q method over the relevant top eQTL per gene.

### Simulation methods

Two types of population-scale scRNA-seq datasets (iPSC Smart-Seq2 and FPP 10X) were simulated using splatPop [55] from splatter v1.17.1 [56]. splatPop uses the gamma-Poisson hierarchical modeling approach from splatter to simulate scRNA-seq data, with eQTL effects incorporated into the baseline gene means for each eGene (y) for each individual (i) as: y_i_ = y_i_ + (y_i_ × G_i_ × w), where G_i_ is the minor allele dosage of the eSNP and w is the eQTL effect size. To ensure realistic simulations, parameters were estimated from real data. Single-cell parameters defining the distribution of library sizes (gamma), common BCV (inverse chi-squared), and dropout rates (logistic) were estimated from the empirical single-cell count data from the donor with the most cells (iPSC Smart-Seq2 donor ID = joxm; FPP 10X donor ID = mita_1). Population parameters defining the distributions of gene mean expression (gamma) and variance in expression (gamma binned by mean expression) were estimated from the mean aggregated expression levels for all genes from either iPSC Smart-Seq2 or FPP 10X. Finally, eQTL parameters defining the distribution of the eQTL effect sizes (gamma) were estimated from the most significant eQTL hit for each gene from bulk iPSC data for eSNPs with MAF > 0.1. The eQTL effects were assigned randomly to 35% (n = 439) of the genes being simulated with eSNPs having a MAF > 0.1 and being within 100 kb of their eGenes. The number of cells to simulate per donor was sampled from a gamma distribution estimated from the number of cells per donor from the empirical data. Data was simulated for genes from chromosome 2 (n = 1255) for 87 individuals in the British cohort for the Smart-Seq2 simulations and for 173 individuals from the European cohorts from 1000Genomes [57]. For the sample size analysis, n individuals were randomly selected from the European cohorts for each simulation (n = 50, 87, 120, 173, 300, 600). For the cells per donor analysis, the rate parameter of the gamma distribution was changed to achieve a range of average number of cells per donor (Smart-Seq2 n = 50, 85, 120, 200, 300, 1000; 10x n = 200, 300, 400, 525, 650, 1000).

To mimic the experimental design of the empirical datasets, cells were simulated in batches with 5 (iPSC Smart-Seq2) or 24 (FPP 10X) individuals per batch, where 28% (iPSC Smart-Seq2) or 14% (FPP 10X) of individuals were replicated in two batches, reflecting the pooling sizes and replication rates in the empirical data. Batch effect sizes were sampled from log-Normal distributions with location = 0.001 and scale = 0.12 (iPSC and FPP) and with the splatPop parameter similarity.scale = 5 (iPSC Smart-Seq2) or similarity.scale = 22 (FPP 10X) set to reflect the batch effects and relationship between samples observed in the real data (Additional file 1: Fig. S5).

The different eQTL mapping methods tested on simulated data were replicated 10 times, with each replicate being an independent splatPop simulation. Due to genetic linkage, the top eSNP mapped for each eGene was not expected to be the simulated eSNP. To account for this, all eQTL hits with an empirical p value below the empirical p value corresponding to the multiple testing threshold determined using Storey Q on the top hits for each gene were considered significant. Each simulated eGene was considered a true positive (TP) if the simulated eSNP was among the significant eSNPs or it was considered a false negative (FN). Note that the FN includes eGenes with no significant eSNPs (type 1) and eGenes with significant eSNPs, but for which the correct eSNP was not significant (type 2). Each simulated gene not assigned an eQTL effect was considered a true negative (TN) if it had no significant eSNPs and a false positive (FP) if it had a significant hit. Performance metrics include power (TP / (TP + FN)), empirical FDR (FP / (FP + TP)), and the Pearson’s correlation between simulated eQTL effect sizes and estimated effect sizes (beta.cor; including only genes simulated as eGenes). Statistical tests were performed in Rv4.0.3 and results are described in detail in Additional file 1: Table S6, Additional file 1: Table S8).

### Aggregation and normalization methods

Single-cell normalization and log2 transformation of normalized counts-per-million were performed on raw single-cell counts using scran (version: 1.14.1) [20] with size factors calculated using the pooled approach described in [21] and a pseudocount of one applied to the log2 transformation to avoid attempting to take the log of zero (i.e., log2(cpm + 1)). The normalized counts were then aggregated using either the mean or the median (Fig. 1). We also considered alternative single-cell normalization techniques, for both Smart-Seq2 and 10X we also used bayNorm (version: 1.8.0) [28] and for 10X we also considered sctransform (version: 0.3.2) [29], but observed minimal differences in normalized expression values (as assessed by Pearson’s correlation at the gene-level over the donor-run combinations, Additional file 1: Table S4) and in eQTL results after dr-mean aggregation (as assessed by overlapping eQTL effect size and p values). Sum aggregation was performed directly on the raw counts (Fig. 1) and followed by pseudo-bulk-like TMM normalization [1] and log2 transformation, as implemented in edgeR (version: 3.28.1) [30].

In all cases, aggregation was performed at two levels of batch (Tables 1 and 2). First, we aggregated all cells from each donor (i.e., d-mean, d-median, d-sum). In this setting, one sample corresponds to one donor (n = 87 in the iPSC Smart-Seq2 dataset, n = 174 in the FPP 10X data; samples with > 5 cells only). Note that all donors from the iPSC Smart-Seq2 dataset had > 5 cells per donor, so this threshold only applied when aggregating over donor run. In the 10X data, only five donors were filtered out by this filter (Additional file 1: Fig. S2c,d). In cases with low sequencing coverage or when poor sequencing quality is a concern, a higher minimum cell threshold may be needed, with the trade-off being fewer donors and thus less power for eQTL discovery [19]. Next, we aggregated separately across donors and sequencing runs (dr-mean, dr-median, dr-sum). In this second setting, one sample is a unique donor-sequencing run combination (when considering samples with > 5 cells, n = 155 in the iPSC Smart-Seq2 data, n = 702 in the FPP 10X data). Visually, the various aggregation methods show a similar picture across donors/samples and genes, with the median aggregations being most affected by the 0-inflated expression (as shown on the iPSC Smart-Seq2 data in Additional file 1: Fig. S14, Additional file 1: Fig. S15).

### Highly variable genes

Highly variable genes (HVGs) were defined as the genes in the top two quartiles based on their squared coefficient of variation (CV^2 = variance / mean^2) calculated across all cells of each different cell-type. In this manner, we identified 21,592 HVGs for the iPSC Smart-Seq2 dataset, and 16,369 for the FPP 10X dataset.

### eQTL mapping strategy

For *cis*-eQTL mapping, we followed Cuomo et al. [13] and adopted a strategy similar to approaches commonly applied in conventional bulk eQTL analyses. We considered common variants (MAF > 10% and Hardy-Weinberg equilibrium P < 0.001) within 100 kb up- and downstream of the gene body. Association tests were performed using linear mixed models (LMMs), fit using LIMIX (version: 2.0.3) [31]. The expression profile of each gene was quantile-normalized to a standard normal distribution and the significance was tested using a likelihood ratio test (LRT).

### Covariates for sc-eQTL mapping

To adjust for experimental batch effects across samples, we included covariates as fixed effects in our LMM models to correct for both known and hidden sources of unwanted variation. These covariates (e.g., batch effects) usually affect the expression of many genes and therefore are detectable in the principal components of expression. Furthermore, global batch effects are orthogonal to the effects of a single *cis* regulatory variant on the expression of one gene, thus accounting for global batch effects in our LMMs will not remove the signal for eQTL. Unless specified otherwise, in our sc-eQTL mapping, we included the first 20 principal components calculated on the relevant expression value aggregation/normalization in the model as fixed effect covariates. However, in order to assess the impact of variations in the types and numbers of covariates included, we also considered alternative methods; first, we considered Probabilistic Estimation of Expression Residuals (PEER (version: 1.3) [38], a method designed specifically to account for global expression confounders in the context of (bulk) eQTL mapping; next, we considered Multi-Omics Factor Analysis (MOFA, which we used in two flavors: with and without sparsity; (version 1.2.0) [38, 40]). While originally designed for multi-omic data, MOFA can be applied when considering a single omic layer (gene expression in this case) to probabilistically identify common sources of variation across all genes and has been applied to a variety of bulk and single-cell datasets. Finally, we tested for this purpose, a method called Linearly Decoded Variational AutoEncoder (LDVAE [39, 40]). This model also aims at finding factors that co-vary across all features (i.e., genes) considered and is especially designed for single-cell data.

All covariates (except LDVAE, see below) were calculated using the relevant aggregated expression values at the level of donor or donor-sequencing run, for all genes tested (63,678 genes). PCA was computed using the R function prcomp. PEER (https://github.com/PMBio/peer/) was run using the R implementation with the number of factors set to 30 and default parameters. MOFA v2 (https://biofam.github.io/MOFA2/) was run using the R implementation, for n = 25 factors. Both default parameters (i.e., MOFA; spikeslab_factors = F, spikeslab_weights = T, ard_factors = F, ard_weights = T) and parameters with the sparsity constraints removed (i.e., MOFA non-sparse; spikeslab_weights = F, ard_weights = F) were used.

Finally, we included a recent approach using variational autoencoders: linearly decoded variational autoencoder (LDVAE; (version scvi-tools: 0.8) [39]). In contrast to the other methods, LDVAE works directly on the single-cell count data. We ran LDVAE following the tutorial (https://www.scvi-tools.org/en/stable/user_guide/notebooks/linear_decoder.html) but changed it to estimate 25 factors over 1000 epochs with 400 warmup epochs. After training, we extracted the LDVAE factors and aggregated the factors over the cells by taking the mean per donor or donor run.

### Mixed effect models

In order to account for possible population substructure in the sample considered as well as, importantly, for repeated observations for the same donor (i.e., across runs, in the “dr” aggregation methods), we include in the linear mixed model a random effect modeling such structure (using a kinship matrix, K):

$$ \mathrm{Y}=\mathrm{covs}+\mathrm{SNP}+{\mathrm{u}}_{\mathrm{K}}+\upvarepsilon, $$

where Y is the expression of the gene considered (aggregated, normalized, and transformed as described above), covs are the expression covariates (described above); SNP is the vector of allele values for the variant being tested; u_K_ is a random variable such that u_K_ ~ N(0, K), where K is a kinship matrix, specifically a SNP-based identity by descent (IBD) matrix estimated using PLINK (version 1.9) [58], and ε is the error term.

Importantly, when considering “dr” aggregation methods (as well as the direct single cell eQTL mapping), this kinship matrix K is expanded to reflect the repeated samples (such that repeated measurements appear as “blocks” along the diagonal).

Additionally, we tested the effect of the inclusion of a noise term in the LMM that accounts for the variable number of cells used to calculate aggregate expression measures, which has been shown to improve eQTL discovery power [14]. Here we also considered adding a term to account for each donor’s sequencing depth (i.e., number of reads per donor, Table 5; Additional file 1: Table S10, Additional file 1: Table S11). Using the LMM framework from LIMIX, we test three models for d-mean to account for different confounders:

$$ \mathrm{Y}=\mathrm{covs}+\mathrm{SNP}+{\mathrm{u}}_{\mathrm{K}}+\upvarepsilon\ \left(\mathrm{baseline},\mathrm{as}\ \mathrm{above}\right) $$

$$ \mathrm{Y}=\mathrm{covs}+\mathrm{SNP}+{\mathrm{u}}_{\mathrm{ncells}}+\upvarepsilon $$

$$ \mathrm{Y}=\mathrm{covs}+\mathrm{SNP}+{\mathrm{u}}_{\mathrm{nreads}}+\upvarepsilon $$

where Y, covs, SNP, u_K_ and ε are defined as above; u_ncells_ is a random variable such that u_ncells_ ~ N(0, diag(1/ncells)), where diag(1/ncells) is a diagonal matrix with the inverse of the number of cells per donor as the diagonal terms and, analogously, u_nreads_ ~ N(0, diag(1/nreads)).

For dr-mean, since we have replicate measurements for each donor (across sequencing runs), we always need to account for population structure (and replicated structure), by including the (expanded) kinship matrix as a random effect. As such, we test the following models:

$$ \mathrm{Y}=\mathrm{covs}+\mathrm{SNP}+{\mathrm{u}}_{\mathrm{K}}+\upvarepsilon\ \left(\mathrm{baseline},\mathrm{as}\ \mathrm{above}\right) $$

$$ \mathrm{Y}=\mathrm{covs}+\mathrm{SNP}+{\mathrm{u}}_{\mathrm{K}+\mathrm{ncells}}+\upvarepsilon $$

$$ \mathrm{Y}=\mathrm{covs}+\mathrm{SNP}+{\mathrm{u}}_{\mathrm{K}+\mathrm{nreads}}+\upvarepsilon $$

Since the LIMIX framework can only account for one random effect, in the latter two models, we introduced a weighting factor (*w*, between 0 and 1) to incorporate the relative weight of the two random effect term matrices, e.g., *w ×* 1/ncells + (1 − *w*) *×* K. We optimized *w* using a grid search per gene (Brent method, [59]), where *w* starts at 0.5 and is varied up or down in steps of 0.1 if the model fit increases with a higher or lower *w*.

### Multiple testing correction

Multiple testing correction for the eQTL results was performed in two steps, as is common practice in eQTL studies [46, 47]. First, to adjust for multiple testing at the gene level (i.e., across SNPs), we used an approximate permutation scheme, analogous to the approach proposed in [46], where, for each gene, we generate 1000 permutations of the genotypes (100 permutations for simulated experiments) while keeping covariates, random effect terms, and expression values fixed. We then adjusted for gene-wise multiple testing using this empirical null distribution. Second, to control for multiple testing across genes, we applied the Storey Q value procedure based on the most significant eQTL per gene, unless otherwise specified. Genes with significant eQTL were reported at an FDR < 5%. This second step is performed after gene selection (i.e., after considering the relevant gene selection in the comparison setting). We deem significant all associations that reach the gene-level corrected p value (step 1) corresponding to the selected gene-level FDR (step 2), i.e., we call as significant all associations to a gene that are below the identified association p value (not just the top associated SNP per gene). We also tested alternatives to the Storey Q value for the second step. In particular, we test (1) the Bonferroni approach (p value< 0.05), which controls the much stricter family-wise error rate (FWER), (2) Benjamini-Hochberg (BH), another commonly used FDR approach; and (3) a recent implementation [41, 42] of the conditional FDR (cFDR), which leverages external data to guide the gene-level multiple testing correction. For the cFDR procedure, we tested using raw association p values from (1) the bulk iPSC associations, (2) GTEx v7 association p values from the EBV transformed lymphocytes, (3) GTEx v7 association p values from tissue meta-analyses results [60], and (4) association p values from a joint eQTL mapping on the mesendoderm and endoderm data from the Cuomo et al. study [13]. Given that the external datasets have imperfect overlap in terms of both SNPs and genes, we limit our focus to overlapping genes and variants for each respective test, unless otherwise specified.

Conditional FDR is a method to condition or transform the p value from one hypothesis test using an external set of test statistics. For a series of hypothesis tests (i = {1, .., n}), P = {P_i_} and Q = {Q_i_} are two sets of random variables, representing the observed p values from the internal (i.e., sc-eQTL; p_i_) and external (e.g., bulk eQTL; q_i_) hypothesis tests, respectively. Traditionally the cFDR is approximated with the empirical joint cumulative distribution function (see Fig. 5a) and is then used to draw a contour through the two-dimensional space of all p value pairs (p, q) = {(p_1_, q_1_), ...(p_n_, q_n_)} that defines the region where H_0_ will be rejected. However, this approach does not explicitly control FDR. Here we apply an adjusted estimator of cFDR proposed and described in detail by Liley and Wallace [42], which was shown to improve power and control the type1 error rate. Briefly, for each p value pair (p_i_, q_i_), the adjusted estimator adds randomly chosen points (p_i’_, q_i’_) to the original p value pair space (p, q). Then the “transformed” p value is calculated as the probability that the randomly chosen points had a more extreme cFDR than the point of interest (p_i_, q_i_). Ultimately, with this approach, we are able to define a better (i.e., a higher TP:FP) H_0_ rejection region than what would be defined by strict thresholds for P and Q, and thus better integrate the external information into the statistical test.

### Replication of bulk eQTL results

Performance of sc-eQTL mapping was measured using two metrics: number of significant sc-eQTL (FDR < 5% unless stated otherwise, described above) and replication with bulk eQTL. A sc-eQTL (i.e., eSNP-eGene pair with FDR < 5% in a single-cell study) was considered to replicate a-bulk eQTL if it passed two criteria: (1) the gene-SNP pair was significant in the bulk test at FDR < 10% and (2) the direction of the effect was consistent between the sc-eQTL and the bulk eQTL.

Note that a small percentage (~ 1%) of non-replicated sc-eQTL in bulk were due to the gene and/or the SNP of interest not being assessed in the bulk eQTL results.

## Supplementary Information

**Additional file 1: Table S1-S4 & S6-S16 and Fig S1-S15.** Supplementary tables and figures including legends.

**Additional file 2: Table S5.** Simulation Smart-Seq2 & 10X QTL mapping results.

**Additional file 3.** Review history.

## Data Availability

The eQTL mapping pipeline is available via: https://github.com/single-cell-genetics/limix_qtl (Apache License 2.0) [61]. splatPop is available in splatter v 1.17.1 (GNU General Public License v3.0) on Bioconductor. The description of how to implement splatPop is available in the package vignette (http://www.bioconductor.org/packages/devel/bioc/vignettes/splatter/inst/doc/splatPop.html). Additional code used for this analysis are available via https://github.com/single-cell-genetics/optimising_singlecell_eqtl_paper (Apache License 2.0) [62]. The HipSci genotype information is made available per sub study and are available via PRJEB11750, EGAS00001000866, EGAS00001002015, EGAS00001002016, EGAS00001002013, EGAS00001002014, EGAS00001002011, EGAS00001002012, EGAS00001002010, EGAS00001002009, EGAS00001002006, EGAS00001001272, EGAS00001002008, EGAS00001001730, EGAS00001001273, EGAS00001002007, EGAS00001002005, and EGAS00001000867. The single-cell iPSC, mesendoderm, and endoderm RNA-seq reads are available via ERP016000 and EGAS00001002278. The bulk iPSC expression data is available per substudy via EGAS00001000529, EGAS00001000593, EGAS00001001137, EGAS00001001318, EGAS00001001727, EGAS00001001986, EGAS00001001987, EGAS00001001988, EGAS00001001989, EGAS00001001990, EGAS00001001991, EGAS00001001992, EGAS00001001993, EGAS00001001994, EGAS00001001995, EGAS00001001996, EGAS00001001997, and ERP007111. The FPP 10X data is available as part of the data published with (Jerber et al., [14]) at: https://zenodo.org/record/4651413. Specifically, we considered the day 11 object only, and subsetted to the cell type of interest: FPP (floor plate progenitors). Finally, bulk and single-cell iPSC count data and checked-out versions of the source code from this manuscript specifically are available at 10.5281/zenodo.4585384 [63].
