# Supplementary material for: Optimizing expression quantitative trait locus mapping workflows for single-cell studies
Source: Genome Biol. 2021 Jun 24;22:188. doi: 10.1186/s13059-021-02407-x (PMC8223300; doi:10.1186/s13059-021-02407-x)
Supplement: Supplementary file 1 — Additional file 1: Table S1-S4 & S6-S16 and Fig S1-S15. Supplementary tables and figures including legends. [file 13059_2021_2407_MOESM1_ESM.docx]

**Supplementary materials**

Optimizing expression quantitative trait locus mapping workflows for single-cell studies

Cuomo, Alvari, Azodi et al.

# Table of contents

Contents

[Table of contents](#_heading=h.gjdgxs) **2**

[Supplementary Tables](#_heading=h.30j0zll) **3**

[Supplementary Figures](#_heading=h.1fob9te) **14**

# Supplementary Tables

**Table S1: Number of eGenes and eQTL replication in bulk for different eQTL mapping methods.** When considering d-mean aggregation (to avoid replicated measurements), and all highly variable genes (20,545 genes tested), we mapped eQTL using a linear mixed model (like the rest of the manuscript), a linear model, and then correlation strategies considering both Pearson and Spearman (see Methods). Replication is assessed using m-bulk calculated in the same way (LMM, LM, Pearson correlation, Spearman correlation respectively) for consistency.

|  | **Discovery** | | **m-bulk replication** | |
| --- | --- | --- | --- | --- |
|  | **eGenes** | **% tested** | **# replicated** | **% replicated** |
| **Linear mixed model** | 1,422 | 6.92% | 854 | 60.06% |
| **Linear model** | 1,447 | 7.04% | 869 | 60.06% |
| **Pearson correlation** | 1,472 | 7.16% | 878 | 59.65% |
| **Spearman correlation** | 1,456 | 7.09% | 834 | 57.28% |

**Table S2: Correlation between normalized expression at donor-run level.** Comparison of both the SmartSeq2 expression levels and 10X expression levels between the different normalization methods used, aggregated at donor-run level and inverse normalized, i.e. the expression levels used in the eQTL mapping. For SmartSeq2 we assessed scran and baynorm, for 10X we assessed scran, baynorm and sctransform.

| **Normalization 1** | **Normalization 2** | **Platform** | **Mean correlation** | **Median  correlation** |
| --- | --- | --- | --- | --- |
| **scran** | **baynorm** | SmartSeq2 | 0.88 | 0.92 |
| **scran** | **baynorm** | 10X | 0.82 | 0.86 |
| **scran** | **sctransform** | 10X | 0.98 | 0.98 |
| **baynorm** | **sctransform** | 10X | 0.85 | 0.89 |

**Table S3:** **Number of eGenes and replication of eQTL for the different aggregation & normalization strategies in Smart-Seq2 iPSC cells.** Considering in the first lines all genes overlapping between each of the methods (n=12,720) and second the highly variable genes (20,335 genes tested; **Methods**) tested (similar to **Table 1**). FDR was controlled at 5% for the discovery and we defined replication as FDR<10% and the same sign in both the matched bulk (N=87, m-bulk) the all bulk set (N=526, a-bulk), and ASE (on the single cell expression data (**Methods**). (tested: test., replicated: repl.)

|  | | | | **m-bulk** | | **a-bulk** | | **ASE replication** | | | |
| --- | --- | --- | --- | --- | --- | --- | --- | --- | --- | --- | --- |
|  | **eGenes** | **#test.** | **%test.** | **#repl.** | **%repl.** | **#repl.** | **% repl.** | **#tested** | **#sign** | **#repl.** | **% repl.** |
| **Gene-level intersection over all methods** | | | | | | | | | | | |
| **dr-mean** | 1,835 | **12,720** | 14.43% | 889 | 48.45% | 1,367 | 74.50% | 1,620 | 1,185 | 506 | 31.23% |
| **dr-median** | 1,337 | **12,720** | 10.51% | 650 | 48.62% | 952 | 71.20% | 1,140 | 825 | 366 | 32.11% |
| **dr-sum** | 1,463 | **12,720** | 11.50% | 819 | 55.98% | 1,153 | 78.81% | 1,280 | 982 | 473 | 36.95% |
| **d-mean** | 1,305 | **12,720** | 10.26% | 768 | 58.85% | 1,046 | 80.15% | 1,144 | 889 | 441 | 38.55% |
| **d-median** | 776 | **12,720** | 6.10% | 470 | 60.57% | 625 | 80.54% | 693 | 545 | 307 | 44.30% |
| **d-sum** | 1,174 | **12,720** | 9.23% | 709 | 60.39% | 951 | 81.01% | 1,023 | 794 | 413 | 40.37% |
| **m-bulk** | 2,590 | **12,720** | 20.36% | - | - | 2,448 | 94.52% | - | - | - | - |
| **Gene-level intersection over mean and sum** | | | | | | | | | | | |
| **dr-mean** | 2,081 | **20,545** | 10.13% | 1,014 | 48.73% | 1,520 | 73.04% | 1,709 | 1,254 | 564 | 33.00% |
| **dr-sum** | 1,598 | **20,545** | 7.78% | 909 | 56.88% | 1,241 | 77.66% | 1,330 | 1,037 | 524 | 39.40% |
| **d-mean** | 1,422 | **20,545** | 6.92% | 854 | 60.06% | 1,123 | 78.97% | 1,187 | 930 | 490 | 41.28% |
| **d-sum** | 1,291 | **20,545** | 6.28% | 778 | 60.26% | 1,018 | 78.85% | 1,069 | 836 | 457 | 42.75% |

**Table S4: Comparison of eQTL mapping power between different single-cell normalization strategies.** eQTL mapping power at donor-mean results for both SmartSeq2 (line 3 and 4, corresponding to **Table 1**, in white) and 10X (line 5-6, corresponding to **Table 3**, in green). For SmartSeq2 12,720 genes are tested, for 10X 3,390 genes are tested. Both eGene discovery power and replication rates of the different tested mappings are very similar. Replication at FDR10% in either matched bulk (SmartSeq2) or GTEx brain (10X), **Methods**.

|  | | **Discovery** | | **m-bulk replication** | | **GTEx replication** | |
| --- | --- | --- | --- | --- | --- | --- | --- |
| **Technology** | **Normalization** | **eGenes** | **% tested** | **# replicated** | **% replicated** | **# replicated** | **% replicated** |
| **SmartSeq2** | | | | | | | |
| SmartSeq2 | Scran | 1,835 | 14.43% | 889 | 48.45% | - | - |
| SmartSeq2 | Baynorm | 1,702 | 13.41% | 864 | 49.76% | - | - |
| **10X** | | | | | | | |
| 10X | Scran | 1,496 | 42.69% | - | - | 205 | 13.70% |
| 10X | Baynorm | 1,152 | 32.88% | - | - | 186 | 16.15% |
| 10X | Sctransform | 1,444 | 41.21% | - | - | 210 | 14.54% |

**Table S6: Detailed statistical test results from the SmartSeq2 simulation analysis.** P-values were adjusted for multiple testing when required using the Bonferroni method. Significant tests (p or p.adj <=0.05) are in bold. DoF: degrees of freedom, reported as DoF numerator / DoF denominator for ANOVA tests.

| **Test** | **Effect** | **DoF** | **test.statistic** | **p** | **p.adj** |
| --- | --- | --- | --- | --- | --- |
| **dependent variable: power** | | | | | |
| repeat measures 2-way ANOVA | level | 1/9 | 9.45 | **0.013** |  |
|  | agg.method | 2/18 | 262.41 | ***4.85e-14*** |  |
|  | level:agg.method | 2/18 | 0.022 | *0.979* |  |
| pairwise t-test | dr vs. d | 29 | 0.918 | *0.366* |  |
| pairwise t-test | mean vs. median | 19 | 11.37 | 6.37e-10 | **1.91e-9** |
|  | mean vs. sum | 19 | 13.77 | 2.43e-11 | ***7.29e-11*** |
|  | median vs. sum | 19 | 0.57 | 0.574 | 1 |
| **dependent variable: empirical FDR** | | | | | |
| repeat measures 2-way ANOVA | level | 1/9 | 15.0467 | ***0.004*** |  |
|  | agg.method | 2/18 | 3.028 | 0.074 |  |
|  | level:agg.method | 2/18 | 0.968 | 0.339 |  |
| pairwise t-test | dr vs. d | 29 | -2.19 | ***0.036*** |  |
| **dependent variable: beta correlation** | | | | | |
| repeat measures 2-way ANOVA | level | 1/9 | 31.386 | ***3.3e-4*** |  |
|  | agg.method | 2/18 | 16.346 | ***0.001*** |  |
|  | level:agg.method | 2/18 | 0.154 | 0.858 |  |
| pairwise t-test | dr vs. d | 29 | 3.824 | ***6.43e-4*** |  |
| pairwise t-test | mean vs. median | 19 | 2.714 | 0.014 | **0.041** |
|  | mean vs. sum | 19 | 6.612 | 2.5e-6 | ***7.5e-6*** |
|  | median vs. sum | 19 | 1.207 | 0.242 | 0.726 |

**Table S7: Number of eGenes for the different aggregation and normalization strategies in 10X midbrain floor plate progenitor cells.** First lines show the eGene discovery in 10X and replication in GTEx brain as well as ASE (3,504 genes, matching Table 3). The second part of the table shows the eQTL results for the in total 10,598 genes (HVGs tested in all methods excluding median). Discovery FDR was controlled at 5%, replication at FDR 10% for both GTEx brain eQTL and ASE.

|  | | | | **GTEx replication** | | **ASE replication** | | | |
| --- | --- | --- | --- | --- | --- | --- | --- | --- | --- |
|  | **eGenes** | **# tested** | **% tested** | **# repl.** | **% repl.** | **#tested** | **# sign** | **# repl.** | **% repl.** |
| **Gene-level intersection over all methods** | | | | | | | | | |
| **dr-mean** | 1,496 | **3,504** | 42.69% | 205 | 13.70% | 1140 | 671 | 126 | 11.05% |
| **dr-median** | 918 | **3,504** | 26.20% | 129 | 14.05% | 684 | 405 | 67 | 9.80% |
| **dr-sum** | 1,041 | **3,504** | 29.71% | 166 | 15.95% | 827 | 514 | 100 | 12.09% |
| **d-mean** | 1,252 | **3,504** | 35.73% | 201 | 16.05% | 1,016 | 635 | 127 | 12.50% |
| **dr-median** | 575 | **3,504** | 16.41% | 115 | 20.00% | 479 | 298 | 65 | 13.57% |
| **dr-sum** | 703 | **3,504** | 20.06% | 150 | 21.34% | 602 | 392 | 94 | 15.61% |
| **Gene-level intersection over mean and sum** | | | | | | | | | |
| **dr-mean** | 3,802 | **10,598** | 35.87% | 647 | 17.02% | 2,262 | 1,383 | 266 | 11.76% |
| **dr-sum** | 3,321 | **10,598** | 31.34% | 612 | 18.43% | 1,802 | 1,156 | 233 | 12.93% |
| **d-mean** | 2,305 | **10,598** | 21.75% | 568 | 24.64% | 1,966 | 1,246 | 255 | 12.97% |
| **d-sum** | 1,830 | **10,598** | 17.27% | 526 | 28.74% | 1,422 | 959 | 222 | 15.61% |

**Table S8: Detailed statistical test results from the 10X simulation analysis.** P-values were adjusted for multiple testing when required using the bonferroni method. Significant tests (p or p.adj <=0.05) are in bold. DoF: degrees of freedom, reported as DoF numerator / DoF denominator for ANOVA tests.

| **Test** | **Effect** | **DFn** | **test.statistic** | **p** | **p.adj** |
| --- | --- | --- | --- | --- | --- |
| **dependent variable: power** | | | | | |
| repeat measures 2-way ANOVA | level | 1/4 | 21.8 | ***0.01*** |  |
|  | agg.method | 2/8 | 960.4 | ***2.96E-10*** |  |
|  | level:agg.method | 2/8 | 0.629 | 0.56 |  |
| pairwise t-test | dr vs. d | 15 | 0.306 | 0.764 |  |
| pairwise t-test | mean vs. median | 10 | 78.27 | 4.59E-14 | ***1.38E-13*** |
|  | mean vs. sum | 10 | 0.99 | 0.344 | 1 |
|  | median vs. sum | 10 | -54.76 | 1.13E-12 | ***3.39E-12*** |
| **dependent variable: empirical FDR** | | | | | |
| repeat measures 2-way ANOVA | level | 1/4 | 2.86 | *0.123* |  |
|  | agg.method | 2/8 | 2.62 | *0.291* |  |
|  | level:agg.method | 2/8 | 1.89 | 0.064 |  |
| **dependent variable: beta correlation** | | | | | |
| repeat measures 2-way ANOVA | level | 1/4 | 7.442 | *0.053* |  |
|  | agg.method | 2/8 | 8.477 | ***0.011*** |  |
|  | level:agg.method | 2/8 | 2.736 | 0.124 |  |
| pairwise t-test | mean vs. median | 9 | 3.83 | 0.004 | **0.012** |
|  | mean vs. sum | 9 | 1.11 | 0.297 | *0.891* |
|  | median vs. sum | 9 | -3.07 | 0.013 | **0.040** |

**Table S9: Increased power in cis-eQTL mapping by correcting for covariates.** Reported are the percentages of number of eGenes (FDR<5%) out of 20,545 genes tested. Limited differences in terms of fraction of eGenes are observed. Highest number of eGenes is found when correcting for 15 PCs in this specific setting and test range.

|  | **5** | **10** | **15** | **20** | **25** |
| --- | --- | --- | --- | --- | --- |
| **PCA** | 8.50% | 9.76% | 10.54% | 10.11% | 9.49% |
| **MOFA sparse** | 6.36% | 8.66% | 8.46% | 8.24% | 8.47% |
| **MOFA non sparse** | 7.96% | 7.67% | 8.06% | 8.11% | 5.07% |
| **PEER** | 8.48% | 9.54% | 10.36% | 10.14% | 10.45% |
| **linear scVI** | 6.28% | 7.07% | 7.28% | 7.72% | 7.96% |

**Table S10:** **Inclusion of random effect to increase discovery power of sc-eQTL mapping in 10X midbrain floor plate progenitor cells.** Shown are the number of eGenes that are discovered at an FDR of 5%. Tested are highly variable genes matched between the two considered aggregations (d-mean and dr-mean, n=10,598).

|  | **Random effect matrix** | | | **Discovery** | |
| --- | --- | --- | --- | --- | --- |
|  | **kinship** | **1/#cells** | **1/#reads** | **eGenes** | **% tested** |
| **d-mean** | ✓ | - | - | 2660 | 25.10% |
| **d-mean** | - | ✓ | - | 2731 | 25.77% |
| **d-mean** | - | - | ✓ | 2737 | 25.83% |
| **dr-mean** | ✓ | - | - | 3167 | 29.88% |
| **dr-mean** | ✓ | ✓ | - | 3140 | 29.63% |
| **dr-mean** | ✓ | - | ✓ | 3317 | 31.30% |

**Table S11:** **Inclusion of random effect to increase discovery power of sc-eQTL mapping in simulations.** Shown are the number of genes discovered at an FDR of 5% that were simulated as eGenes (# True Positives; TPs) or not (# False Positives; FPs) across the 10 simulation replicates for each data type (ss2: SmartSeq2). The change (Δ) indicates the number of additional TPs (blue) and FPs (red) with the inclusion of the sampling variation in the random effects matrix compared to using the kinship alone. Tested are all simulated genes (n = 1255, where 439 are simulated as eGenes). SmartSeq2: ss2.

| **Type** | **Level** | **Random Effect Matrix** | | | **# True Positives** | | | **# False Positives** | | |
| --- | --- | --- | --- | --- | --- | --- | --- | --- | --- | --- |
|  |  | **kinship** | **1/#cells** | **1/#reads** | **mean** | **stdev** | **Δ** | **mean** | **stdev** | **Δ** |
| ss2 | d-mean | ✓ | - | - | 200.4 | 11.1 |  | 16.6 | 4.1 |  |
| ss2 | d-mean | - | ✓ | - | 204.5 | 9.3 | 4.1 | 18.1 | 4.6 | 1.5 |
| ss2 | d-mean | - | - | ✓ | 204.3 | 8.5 | 3.9 | 19.1 | 7.8 | 2.5 |
| ss2 | dr-mean | ✓ | - | - | 204.7 | 8.0 |  | 16.3 | 2.8 |  |
| ss2 | dr-mean | ✓ | ✓ | - | 215.1 | 9.3 | 10.4 | 26.6 | 6.3 | 10.3 |
| ss2 | dr-mean | ✓ | - | ✓ | 219.1 | 9.7 | 14.4 | 36.1 | 11.3 | 19.8 |
| 10X | d-mean | ✓ | - | - | 91.7 | 8.0 |  | 10.1 | 2.9 |  |
| 10X | d-mean | - | ✓ | - | 105.1 | 10.8 | 13.4 | 11.9 | 2.9 | 1.8 |
| 10X | d-mean | - | - | ✓ | 104.6 | 10.0 | 12.9 | 12.0 | 2.4 | 1.9 |
| 10X | dr-mean | ✓ | - | - | 97.5 | 8.6 |  | 13.4 | 4.8 |  |
| 10X | dr-mean | ✓ | ✓ | - | 114.6 | 9.0 | 17.1 | 25.2 | 5.6 | 11.8 |
| 10X | dr-mean | ✓ | - | ✓ | 114.3 | 9.5 | 16.8 | 21.6 | 4.7 | 8.2 |

**Table S12:** **Conditional FDR increases eGene discovery in iPSC Smart-Seq2 data while replication fractions stay consistent.** Shown are the number of eGenes that are discovered at an FDR of 5% of the resective method (p <0.05 for Bonferroni), and the replication in all bulk (a-bulk) defined as FDR <10% and same sign. Tested are all genes expressed when considering the mean aggregation/normalization method and that are expressed in bulk (n=20,334).

|  | **Discovery (FDR 5%)** | | **all bulk all (FDR 10%)** | |
| --- | --- | --- | --- | --- |
|  | **eGenes** | **% tested** | **# replicated** | **% replicated** |
| **cFDR** | 2887 | 14.20% | 1990 | 68.93% |
| **Storey Q** | 2058 | 10.12% | 1425 | 69.24% |
| **BH** | 2028 | 9.97% | 1410 | 69.53% |
| **Bonferroni** | 512 | 2.52% | 424 | 82.81% |

**Table S13: Conditional FDR based on external datasets increases eQTL discovery power.** Shown are the number of eGenes that are discovered at an (c)FDR of 5% of the respective method (p < 0.05 for Bonferroni), and the replication in all bulk (a-bulk) defined as FDR <10% and same sign. For comparison between the different reference sets we matched the genes between the different datasets considering only the genes that are expressed in all four datasets (n=13,653).

|  | **Discovery (FDR 5%)** | | **all bulk all (FDR 10%)** | |
| --- | --- | --- | --- | --- |
|  | **eGenes** | **% tested** | **# replicated** | **% replicated** |
| **cFDR (cell type agnostic scQTL)** | 1898 | 13.90% | 1482 | 78.08% |
| **cFDR (GTEx metasoft RE2)** | 1859 | 13.62% | 1459 | 78.48% |
| **cFDR (GTEx metasoft FE)** | 1810 | 13.26% | 1426 | 78.78% |
| **cFDR (closest tissue GTEx)** | 1729 | 12.66% | 1364 | 78.89% |
| **cFDR (GTEx metasoft RE)** | 1720 | 12.60% | 1352 | 78.60% |
| **Storey Q** | 1413 | 10.35% | 1119 | 79.19% |
| **BH** | 1413 | 10.35% | 1119 | 79.19% |
| **Bonferroni** | 434 | 3.18% | 364 | 83.87% |

**Table S14: Conditional FDR based on external datasets increases eQTL discovery power.** Shown are the number of eGenes that are discovered at an cFDR of 5% when using the respective reference set (i.e. number of genes considered is different for every cFDR run), and the replication in all bulk (a-bulk) defined as FDR <10% and same sign.

|  |  | **Discovery (FDR 5%)** | | **all bulk all (FDR 10%)** | |
| --- | --- | --- | --- | --- | --- |
|  | Genes tested | **eGenes** | **% tested** | **# replicated** | **% replicated** |
| **cFDR (cell type agnostic scQTL)** | 20,540 | 2,821 | 13.73% | 1,852 | 65.65% |
| **cFDR (LCL GTEx)** | 16,491 | 2,183 | 13.24% | 1,513 | 78.99% |
| **cFDR (GTEx metasoft RE2)** | 15,550 | 2,028 | 13.04% | 1,602 | 78.99% |
| **cFDR (GTEx metasoft FE)** | 15,550 | 1,974 | 12.69% | 1,567 | 79.38% |
| **cFDR (GTEx metasoft RE)** | 15,550 | 1,881 | 12.10% | 1,489 | 79.16% |

**Table S15: Effects of different optimisation strategies and their combination on the power to map eQTL in single-cell data.** Shown are the number of eGenes that are discovered at FDR<5% of the respective method (FWER 0.05 for Bonferroni), and the replication in all bulk (a-bulk) defined as FDR<10% and same sign. Tested are genes expressed when considering the d-mean aggregation/normalization method and that are expressed in bulk (n=20,334).

| **Settings** | | **Discovery (FDR 5%)** | | **all bulk all (FDR 10%)** | |
| --- | --- | --- | --- | --- | --- |
| **Mapping** | **Mult-test** | **eGenes** | **% tested** | **# replicated** | **% replicated** |
| **dr-mean SV** | cFDR | 3,592 | 17.66% | 2,309 | 64.28% |
| **dr-mean SV** | Storey Q | 2,855 | 14.04% | 1,823 | 63.85% |
| **dr-mean SV** | BH | 2,681 | 13.18% | 1,750 | 65.27% |
| **dr-mean SV** | Bonferroni | 609 | 2.99% | 504 | 82.76% |
| **dr-mean** | cFDR | 2,887 | 14.20% | 1,990 | 68.93% |
| **dr-mean** | Storey Q | 2,055 | 10.11% | 1,423 | 69.25% |
| **dr-mean** | BH | 2,028 | 9.97% | 1,410 | 69.53% |
| **dr-mean** | Bonferroni | 510 | 2.51% | 423 | 82.94% |
| **d-mean** | cFDR | 1,879 | 9.24% | 1,624 | 78.38% |
| **d-mean** | Storey Q | 1,402 | 6.89% | 1,067 | 76.11% |
| **d-mean** | BH | 1,262 | 6.21% | 977 | 77.42% |
| **d-mean** | Bonferroni | 340 | 1.67% | 285 | 83.82% |

**Table S16: Effects of different optimisation strategies and their combination on the power to map eQTL in single-cell data.** Similar to Table 8, but considering FDR<1%**.** Shown are the number of eGenes that are discovered at an FDR of 1% of the respective method (P 0.05 for Bonferroni), and the replication in all bulk (a-bulk) defined as FDR <10% and same sign. Tested are genes expressed when considering the d-mean aggregation/normalization method and that are expressed in bulk (n=20,334).

| **Settings** | | **Discovery (FDR 5%)** | | **all bulk all (FDR 10%)** | |
| --- | --- | --- | --- | --- | --- |
| **Mapping** | **Mult-test** | **eGenes** | **% tested** | **# replicated** | **% replicated** |
| **dr-mean SV** | **cFDR** | 2,140 | 10.52% | 1,615 | 75.47% |
| **dr-mean SV** | **Storey Q** | 1,606 | 7.90% | 1,195 | 74.41% |
| **dr-mean SV** | **BH** | 1,550 | 7.62% | 1,162 | 74.97% |
| **dr-mean SV** | **Bonferroni** | 473 | 2.33% | 396 | 83.72% |
| **dr-mean** | **cFDR** | 1,798 | 8.84% | 1,405 | 78.14% |
| **dr-mean** | **Storey Q** | 1,283 | 6.31% | 985 | 76.77% |
| **dr-mean** | **BH** | 1,274 | 6.27% | 981 | 77.00% |
| **dr-mean** | **Bonferroni** | 407 | 2.00% | 336 | 82.56% |
| **d-mean** | **cFDR** | 1,284 | 6.31% | 1,077 | 83.88% |
| **d-mean** | **Storey Q** | 893 | 4.39% | 716 | 80.18% |
| **d-mean** | **BH** | 829 | 4.08% | 666 | 80.34% |
| **d-mean** | **Bonferroni** | 251 | 1.23% | 205 | 81.67% |

# Supplementary Figures

***
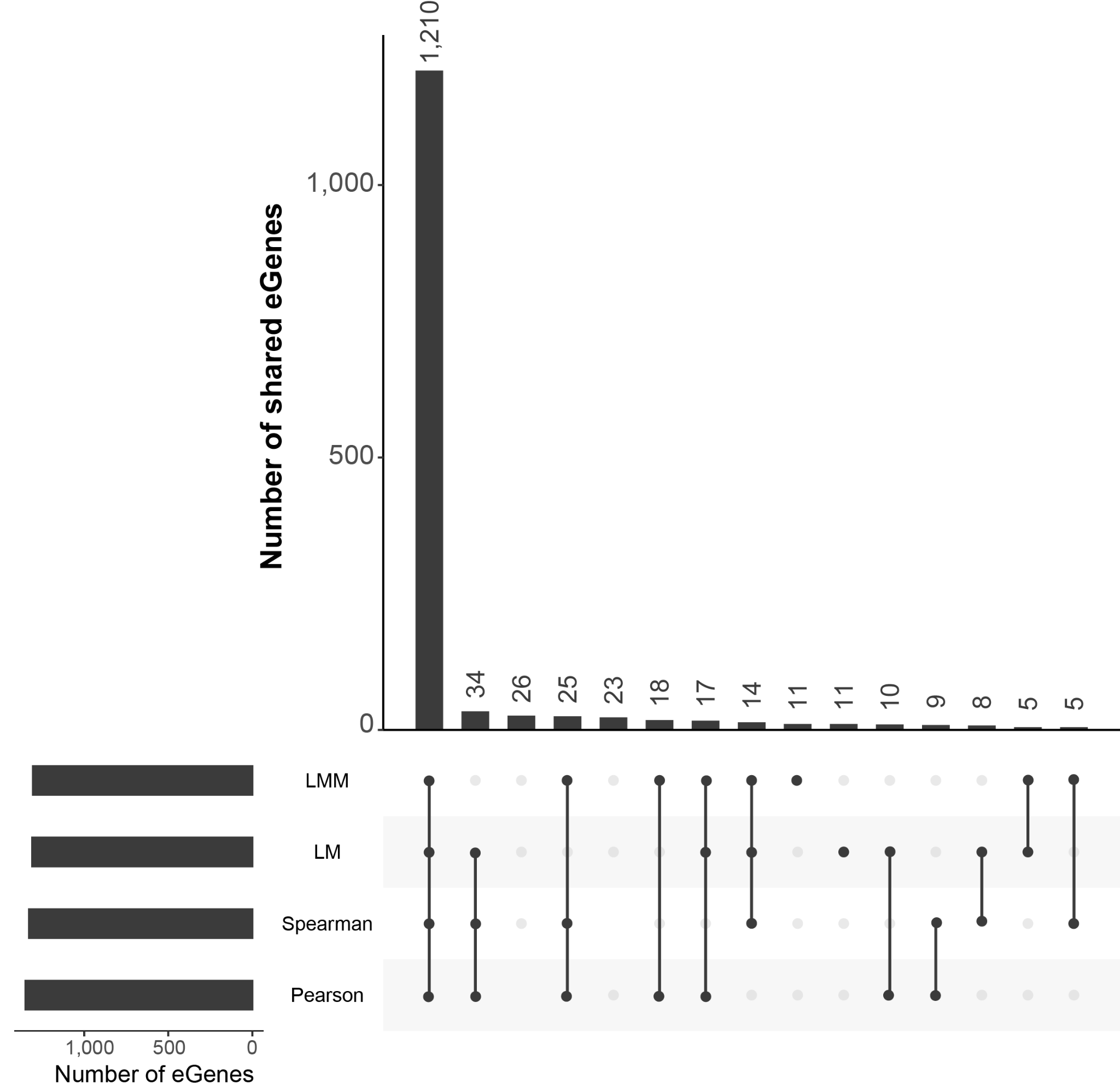
***

**Fig S1: Comparison of eGene discovery across eQTL mapping methods.** Overlap of eGenes identified using a Linear Mixed Model vs Linear Model vs a Spearman correlation versus a Pearson correlation. A large degree of eGenes is shared between the different methods, highlighting that the method used, in the context of non-repeated donors (d-mean), has limited influence on eGene discovery power.


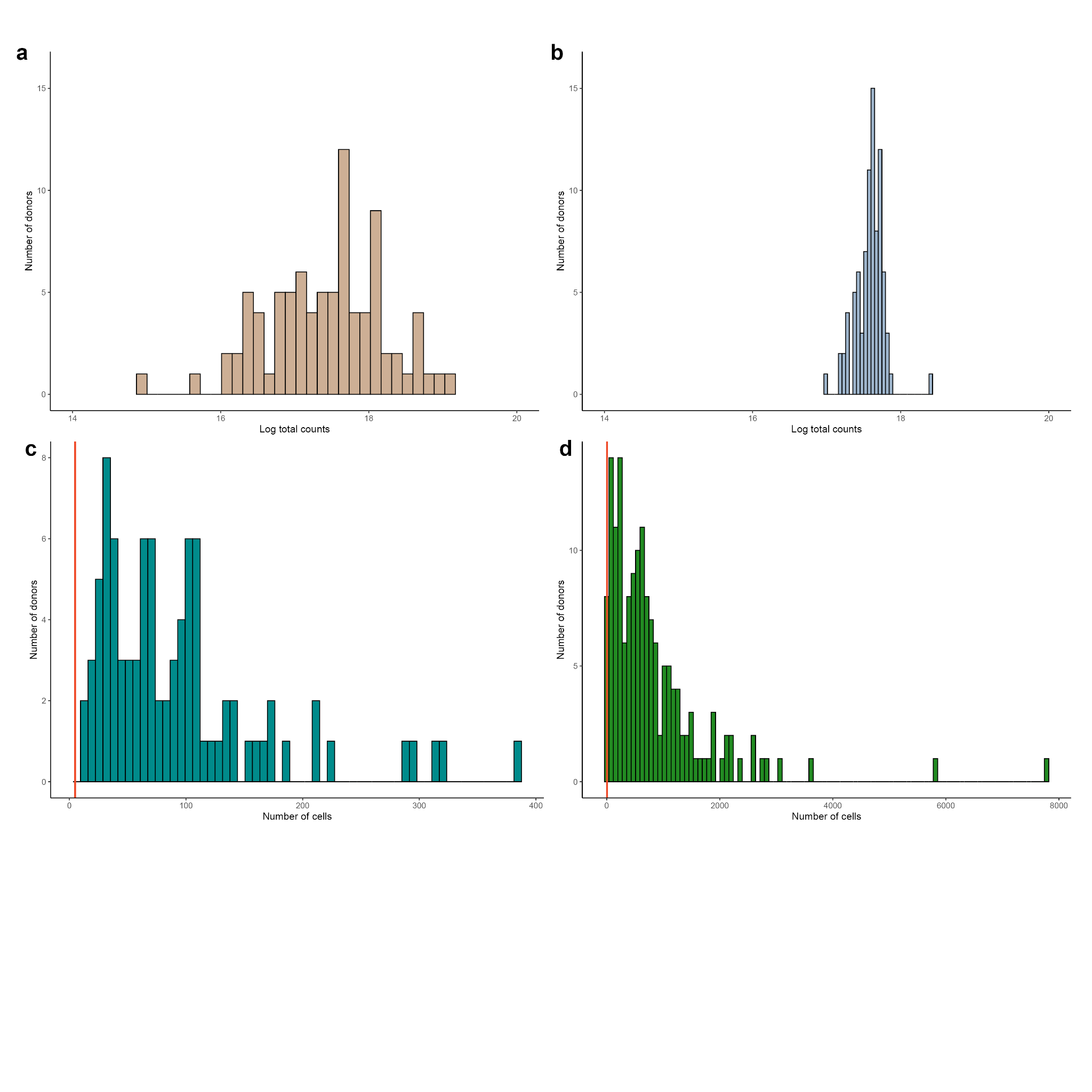


**Fig S2: Distribution of reads and cells across donors.**

Histograms representing the distribution of total reads (genome-wide) per individual for matched iPSC data (from the same 87 individuals) using a) single cell SmartSeq2 and b) bulk (right) RNA-seq data. c) Histogram representing the distribution of numbers of cells across donors in iPSC SmartSeq data. d) As in c), but considering the FPP 10X data. The red vertical line indicates the 5-cell used threshold, which removes 0 donors in the iPSC SmartSeq2 data (panel c), and five donors (5/174) in the FPP 10X data (panel d).


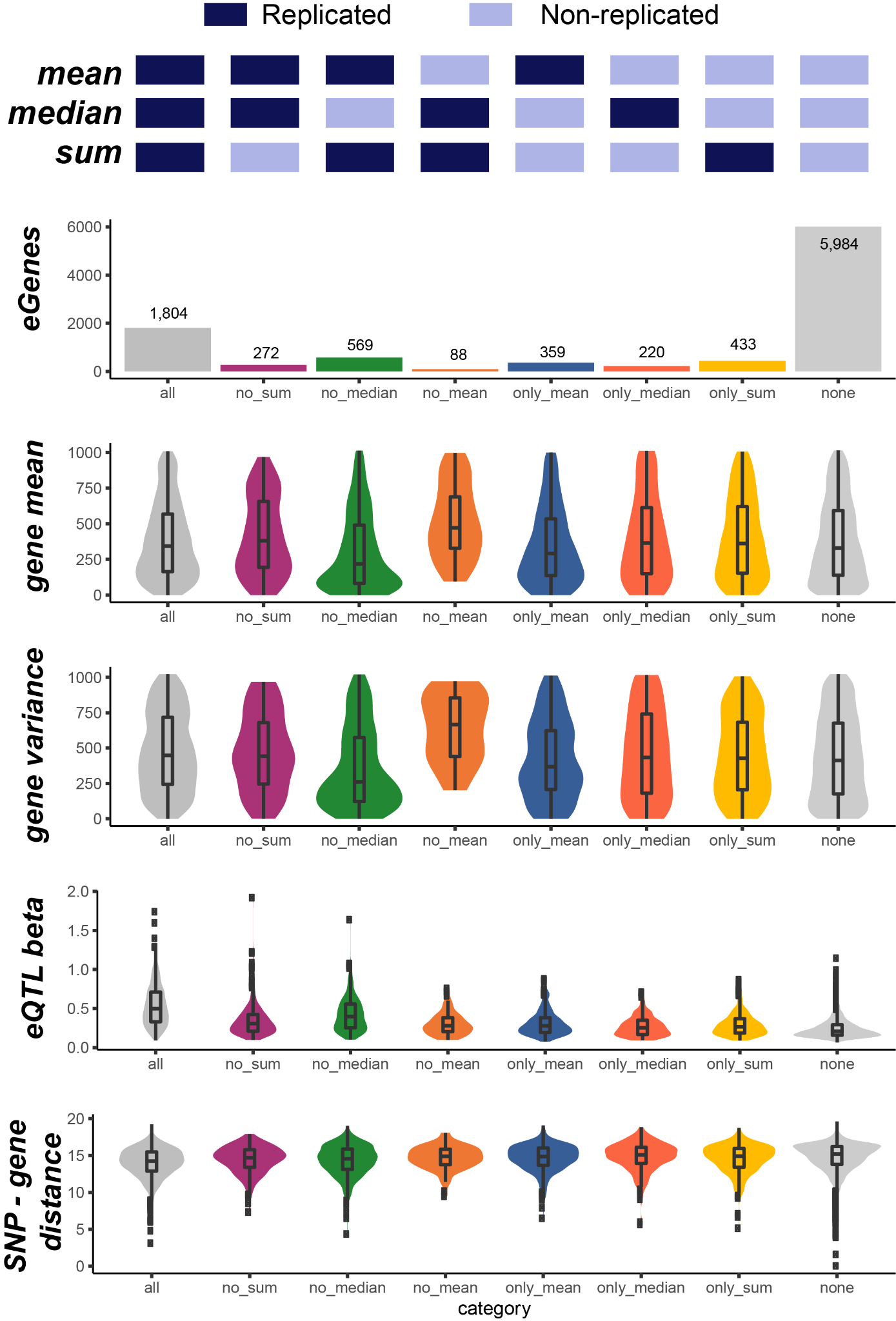


**Fig S3: Properties of bulk eQTL replicated using single-cell data.** Distributions of various properties of iPSC bulk eGenes and eQTL replicated using single-cell expression. When considering a-bulk results and ‘dr’ aggregations (mean, median and sum), bar plots represent number of eQTL replicated in each category, then distributions are shown for the eGenes’ mean and variance (using bulk expression data), for the eQTL effect size (in the bulk results) and for the distance between the eGene and the eQTL SNP.


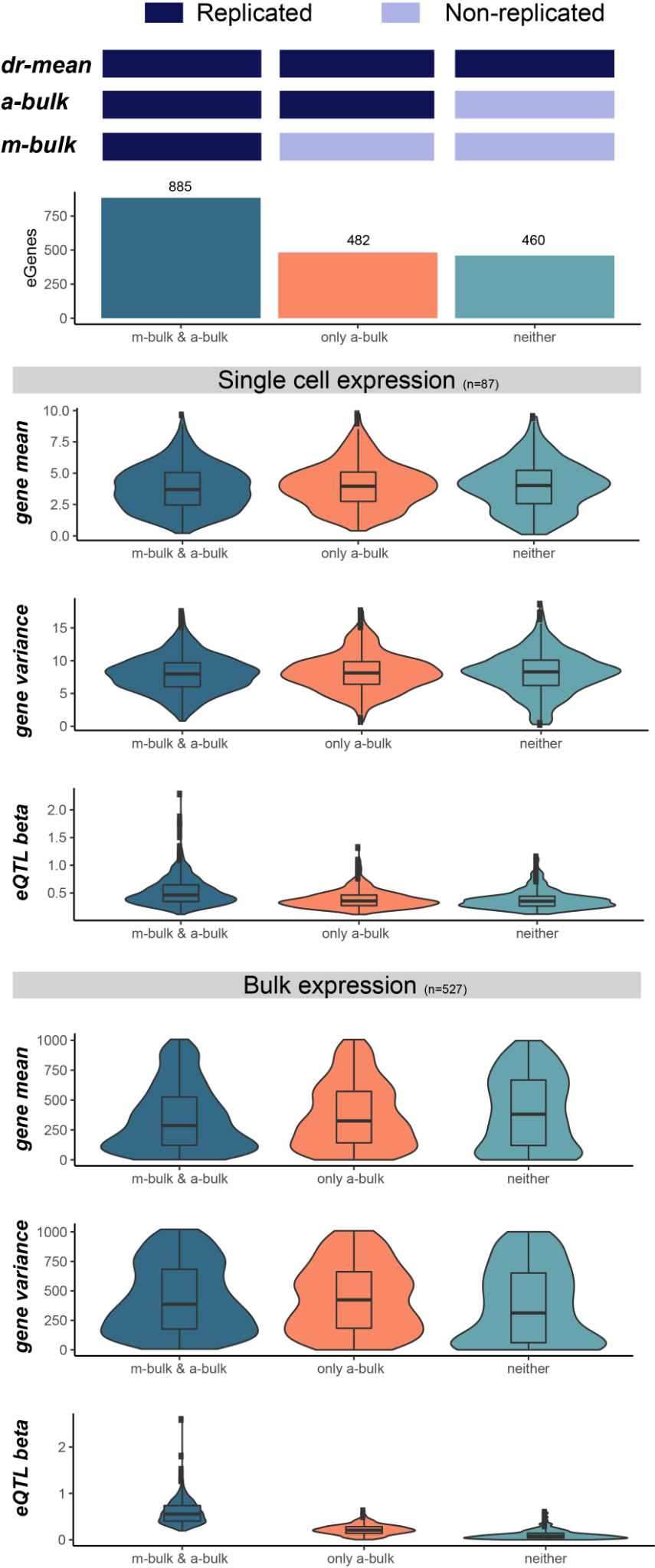


**Fig S4: Properties of sc-eQTL replicated using bulk expression data.** Similar to Figure S3, but this time considering single-cell eQTL (using dr-mean) and whether they could be replicated in both a-bulk and m-bulk, only one of the two, or neither. The distributions of eGenes’ mean and variance, and the eQTL effect size are shown both for single-cell and bulk expression data.


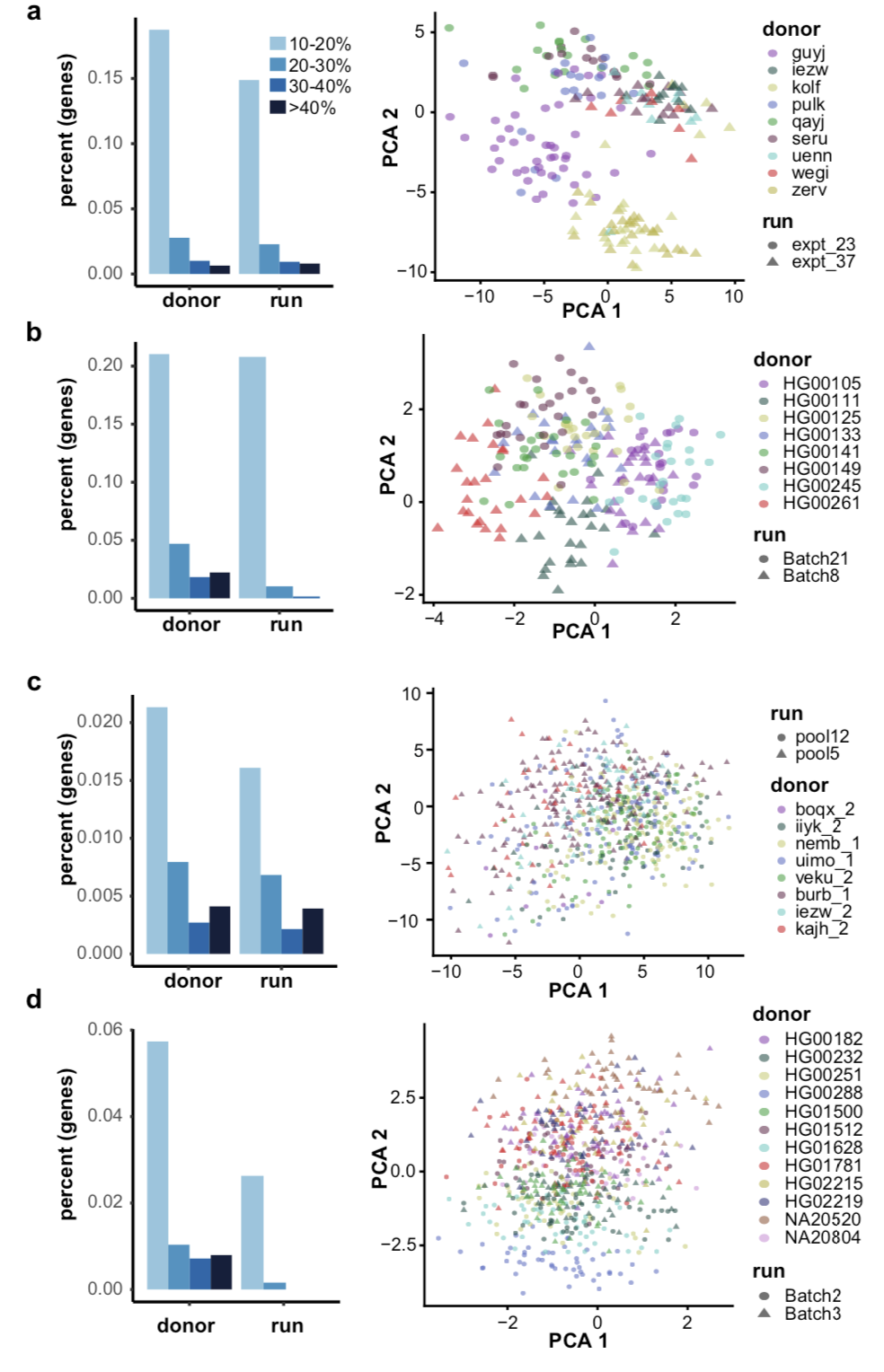


**Fig S5: Empirical and simulated population characteristics.** iPSC SmartSeq2 **(a)** empirical and **(b)** simulated data and NeuroSeq 10X **(c)** empirical and **(d)** simulated data. Barplots (left) show the percent of genes with differing degrees (color) of variance explained by the donor and run factors (x-axis). PCA plots (right) show a subset of cells for samples from two experimental runs, with cells shaped by run and coloured by donor.


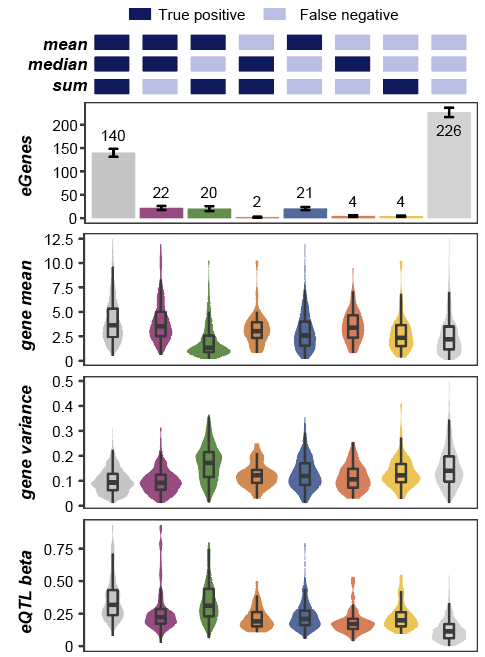


**Fig S6: Properties of SmartSeq2-iPSC simulated eQTL correctly mapped by different aggregation methods.** The bar graph shows the number of eQTL correctly mapped by the set of aggregation methods (top) at the donor-run level (or not identified by any method; gray). Below are the distributions of simulated eGene mean, eGene variance, and eQTL effect size for each set of eQTL (similar to S3).

***
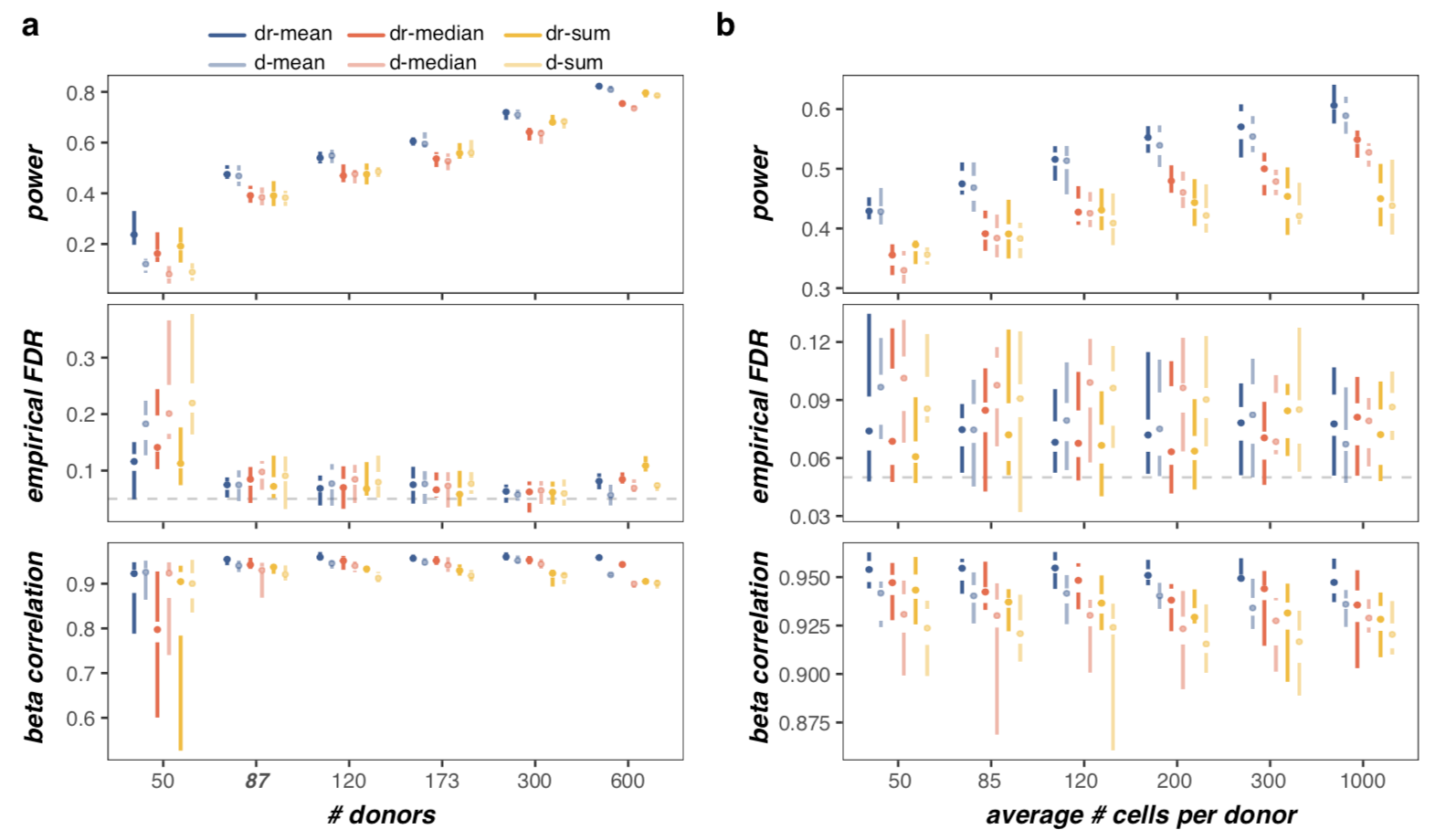
***

**Fig S7: Tufte’s boxplots of (top) power, (middle) empirical FDR, and (bottom) beta correlation for eQTL mapping performance on simulated iPSC Smartseq2 populations.** Performance metrics are as described in Fig. 3. (a) Performance results on datasets simulated with different numbers of donors. Donors were sampled randomly for each of the 10 replicates. (b) Performance results on datasets simulated to have different numbers of cells per donor (per run). The number of cells for each donor-run was sampled from a gamma distribution parameterized from the empirical iPSC Smartseq2 cells per donor-run (shape = 2.1, rate=0.025) which results in a mean of 85 cells per donor-run. Other mean # cells/donor-run were achieved using the following rate parameters (50=0.04, 120=0.017, 200=0.0105, 300=0.007, 1000=0.0021). Colors as in Fig. 2. Shade designates aggregation level (donor-run: dark, left; donor: light, right). The point indicates the median, the gap indicates the interquartile range, and lines indicate the whiskers.

| 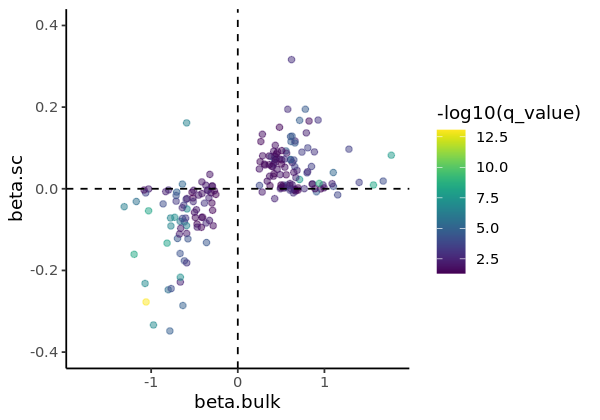 | 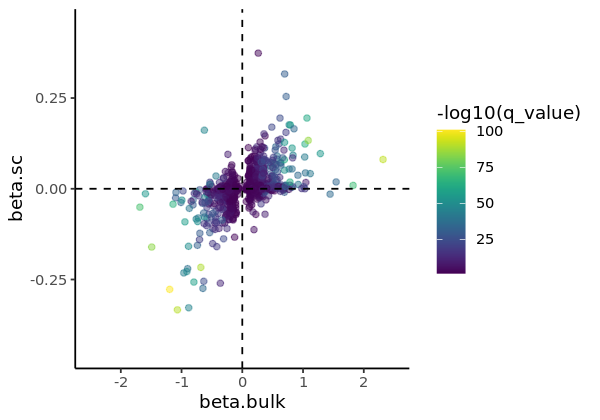 |
| --- | --- |
| 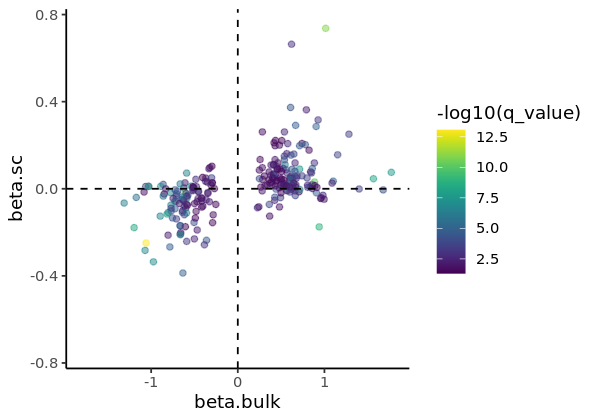 | 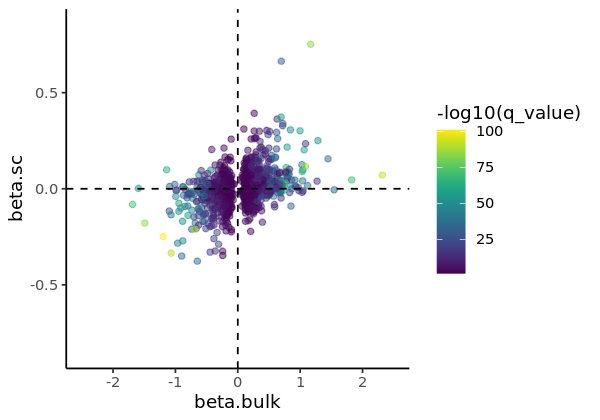 |

**Fig S8: eQTL mapping results of mapping eQTL directly on single-cell expression data.** Comparison of effect sizes between single-cell (y-axis) and bulk (x-axis) eQTL mapping results, when using individual cells as observations. Top: using all cells, bottom using only 5 cells per donor. Left: m-bulk, right a-bulk. Points are coloured by the q-value in the bulk results.


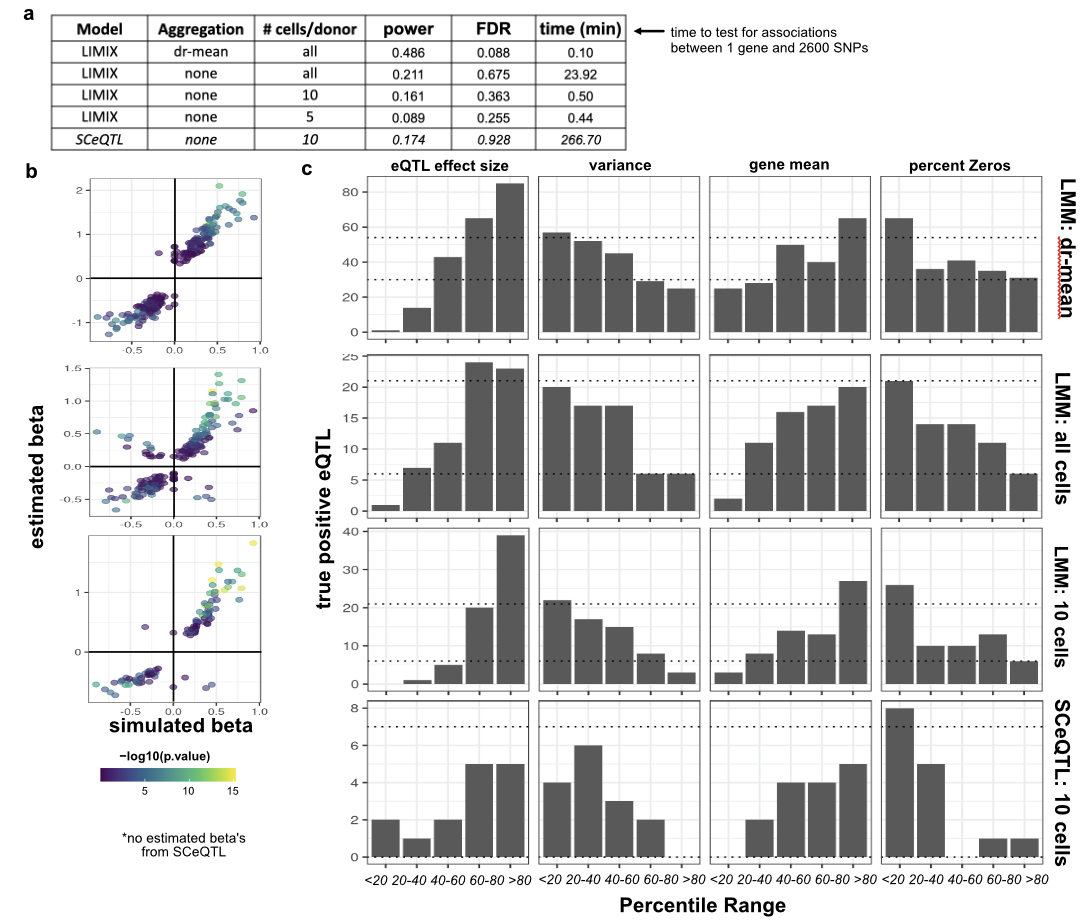


**Fig S9: Results of mapping eQTL directly on simulated single-cell expression data.** (a) Performance comparison of mapping methods on one simulated iPSC dataset (87 donors, average 85 cells per donor). The linear mixed models (LMMs) were implemented in LIMIX. (b) The estimated (y-axis) and simulated (x-axis) eQTL effect sizes for genes called as eGenes by the respective model. Points are colored by the empirical p.value. (c) Number of true positive eQTL from each percentile range for simulated eQTL effect size, coefficient of variation, gene mean, and percent zeros. Dashed lines represent the 5th and 95th percentile range of the expected random distribution.


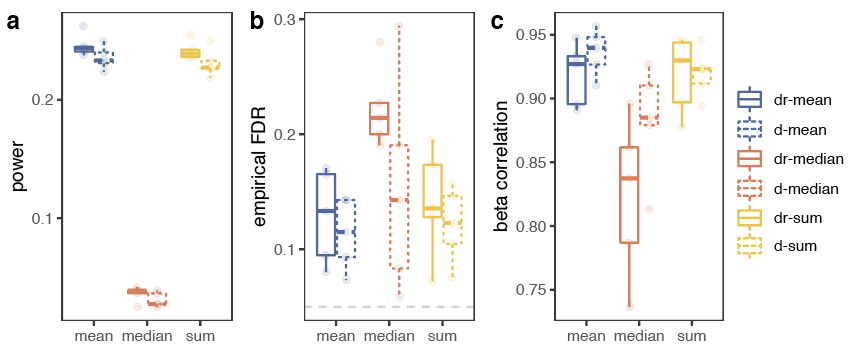


**Fig S10: Summary of eQTL mapping performance on simulated 10X neuron differentiation datasets. (a)** Power to detect simulated eQTL (# true positives / # simulated eQTL). **(b)** Empirical FDR (false discovery rate at FDR < 5%). **(c)** Pearson’s correlation between the ground truth and estimated effect sizes for genes simulated as eGenes. Colors and line types are as in Fig. 2. Box plots summarize the distribution, while the points show performance for each replicate (n=10).

***
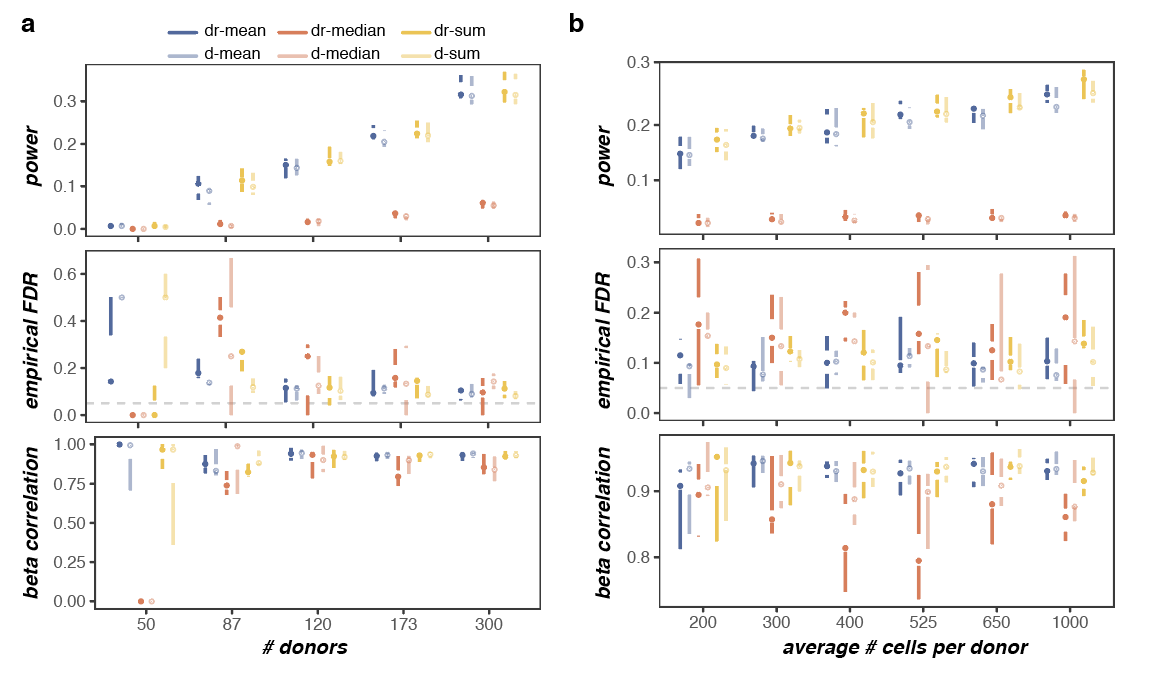
***

**Fig S11: Tufte’s boxplots of (top) power, (middle) empirical FDR, and (bottom) beta correlation for eQTL mapping performance on simulated 10x neuro differentiation populations.** Performance metrics are as described in Fig. 3. (a) Performance results on datasets simulated with different numbers of donors. Donors were sampled randomly for each of the 10 replicates. (b) Performance results on datasets simulated to have different numbers of cells per donor (per run). The number of cells for each donor-run was sampled from a gamma distribution parameterized from the empirical iPSC Smartseq2 cells per donor-run (shape = 1.23, rate=0.00235) which results in a mean of 525 cells per donor-run. Other mean # cells/donor-run were achieved using the following rate parameters (200=0.006, 300=0.004, 400=0.003, 525=0.00234, 650=0.0019, 1000=0.00122). Colors as in Fig. 2. Shade designates aggregation level (donor-run: dark, left; donor: light, right). The point indicates the median, the gap indicates the interquartile range, and lines indicate the whiskers.

**
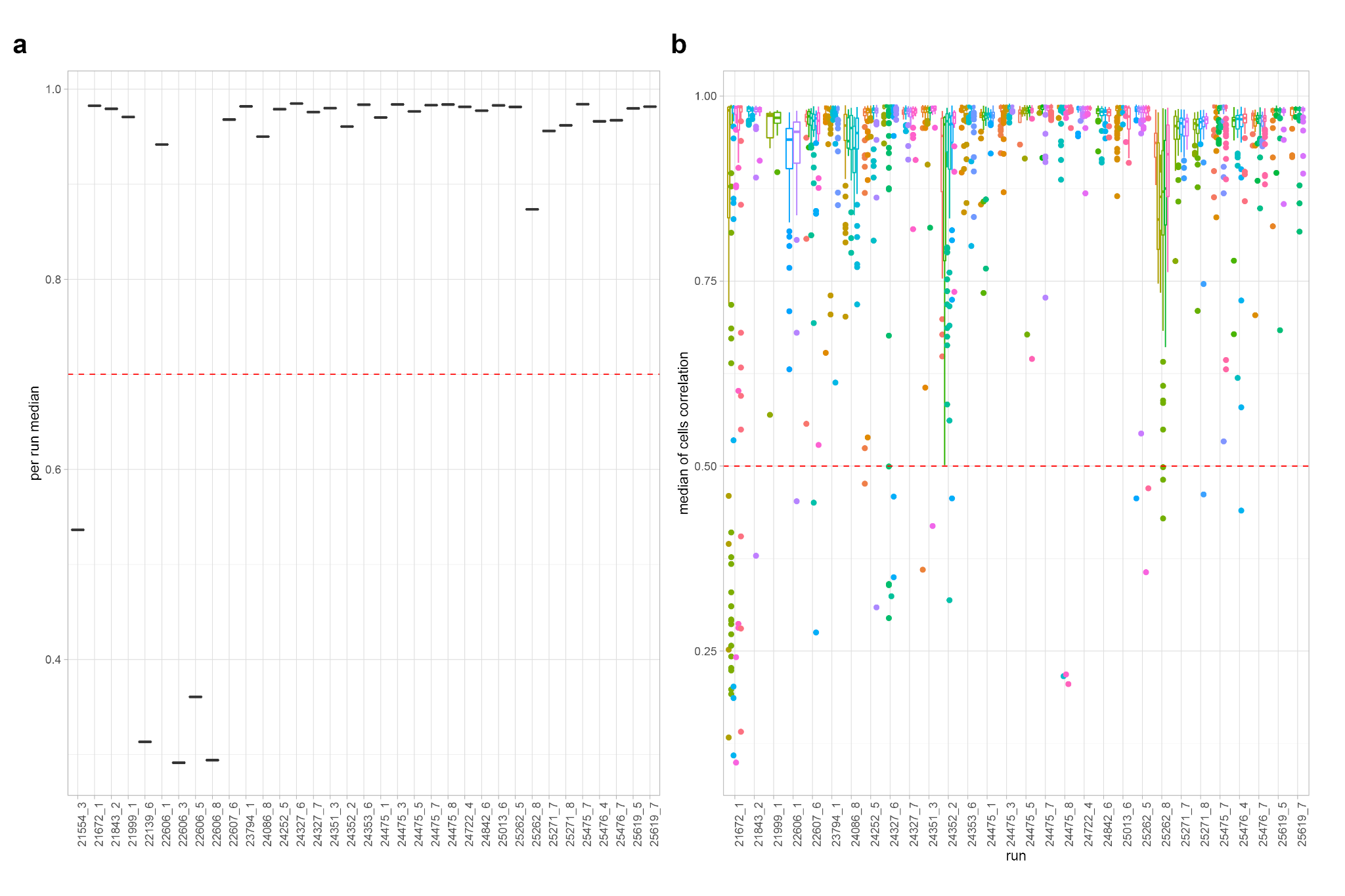
**

**Fig S12: Correlation-based cell QC.** (**a**) Median cell-cell correlation calculated per run. 1) For each cell, we calculated its median (Pearson’s) correlation with all other cells; 2) for each run, we calculated the median of these resulting median correlations; finally 3) runs with median cell-cell correlation < 0.7 (red dotted line) were discarded. (**b**) For the remaining cells, median cell-cell correlations were re-calculated, and cells with median cell-correlation < 0.5 (red dotted line) were discarded.

***
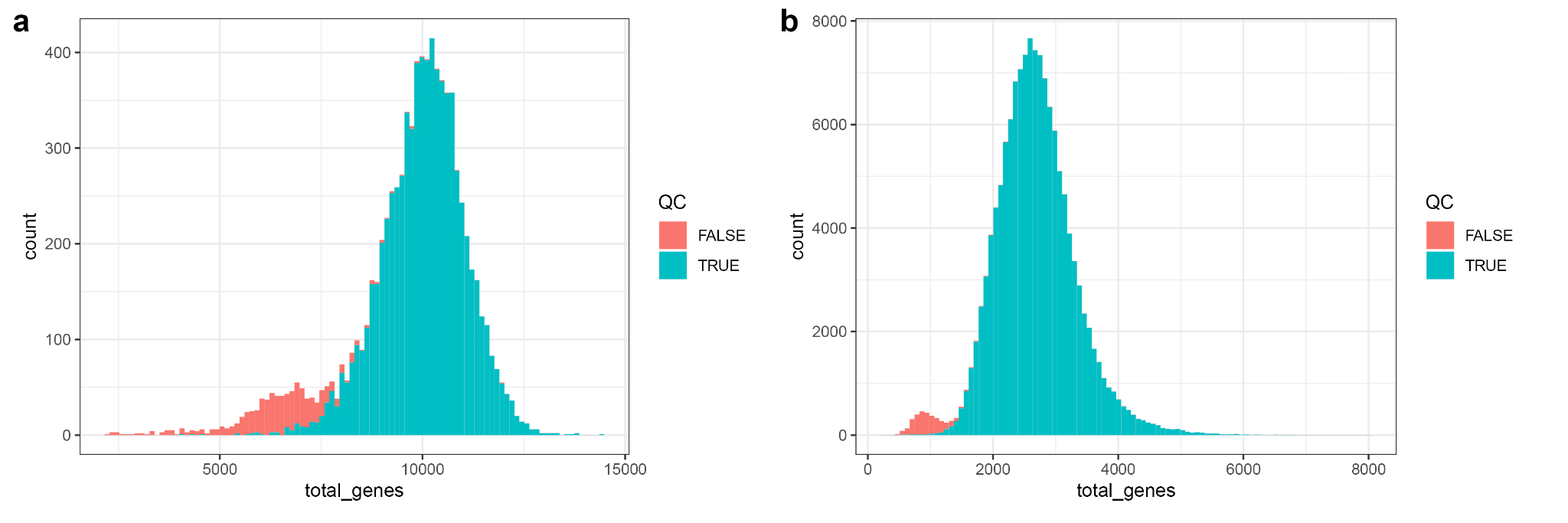
***

**Fig S13: Cell QC.** Validation of the cell QC strategy used in this study (as described in Methods) using more a traditional cell QC metric. When considering the total number of genes detected in each cell as an example of a standard metrics QC across cells, we observe that both for the iPSC SmartSeq2 data (panel a) and the FPP 10X data (panel b) the cells discarded using our cell QC also represent outlying cell sub-populations when considering this metric.


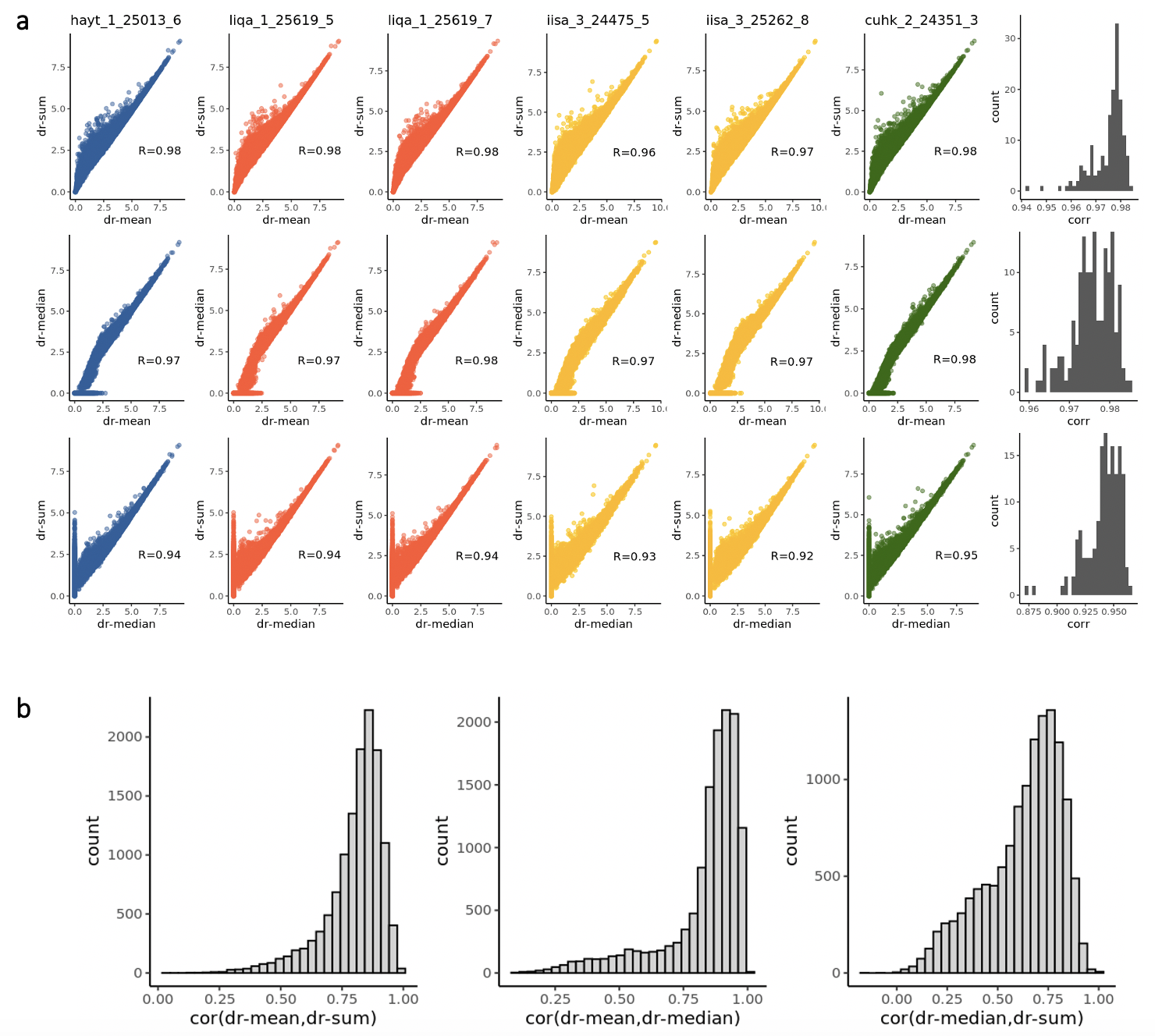


**Fig S14: Comparison of ‘dr’ aggregated measures. (a)** For a random selection of 4 donors (two of which present in two sequencing runs, resulting in 6 donor-run combinations, or samples), scatter plots between aggregation metrics, across the set of common genes (n=12,720). First row is dr-mean vs dr-sum, second dr-mean vs dr-median, third dr-median vs dr-sum. The last column represents, for each of the comparisons a histogram of the distribution of correlations, across donors. **(b)** Histograms representing the distribution of correlations across donors, for each genes, for the same three comparisons as in **(a).**

**
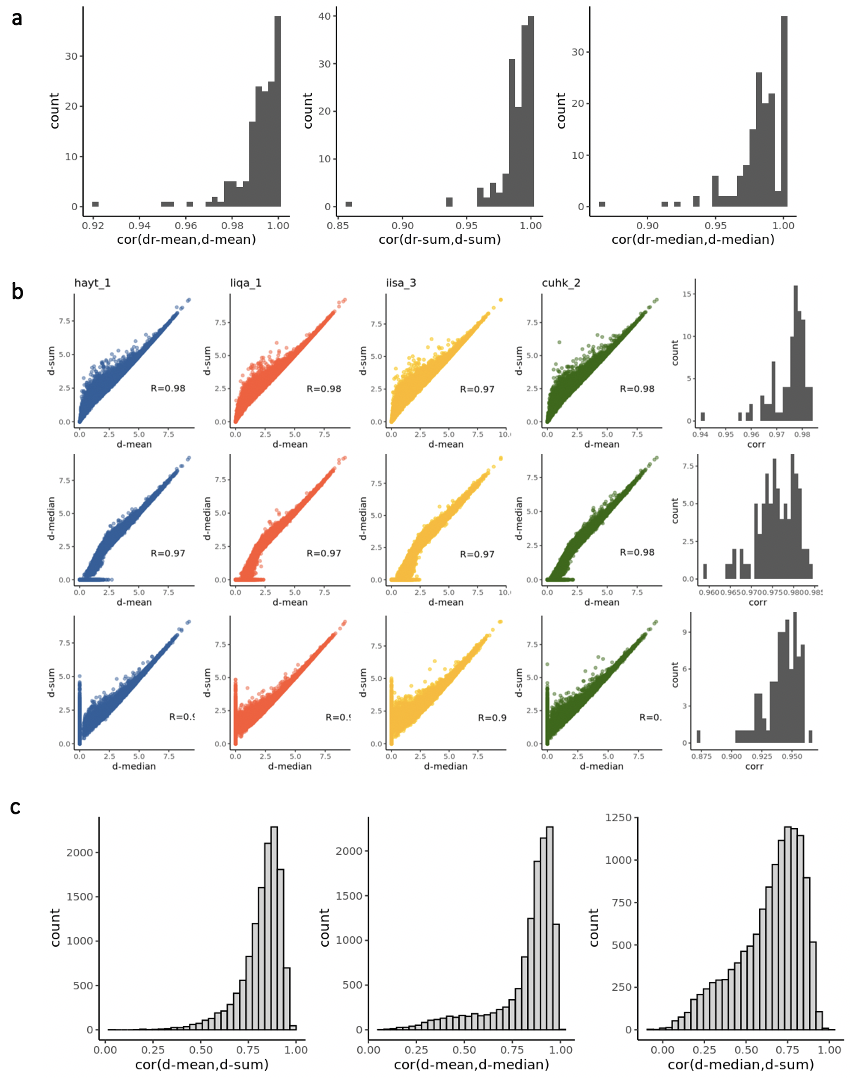
**

**Fig S15: Comparison of ‘d’ aggregated measures. (a)** Histograms of correlations between ‘dr’ and ‘d’ aggregation measures, for each of mean, sum, median. **(b,c)** Similar to Supplementary Fig. S14, panels a and b, but across ‘d’ aggregation methods (instead of ‘dr’; the same donors are considered).
