# Supplementary material for: Optimizing expression quantitative trait locus mapping workflows for single-cell studies
Source: Genome Biol. 2021 Jun 24;22:188. doi: 10.1186/s13059-021-02407-x (PMC8223300; doi:10.1186/s13059-021-02407-x)
Supplement: Supplementary file 3 — Additional file 3. Review history. [file 13059_2021_2407_MOESM3_ESM.docx]

Review History

**First round of review**

**Reviewer 1**

**Are you able to assess all statistics in the manuscript, including the appropriateness of statistical tests used?**

Yes, and I have assessed the statistics in my report.

**Comments to author:**

Conducting eQTL analysis using scRNA-seq data is a promising direction for studying gene regulation at single-cell resolutions. This paper addresses one possible ways of doing this task: collecting single-cell data of the same cell type and composing pseudo-bulk gene expression data for cells of the same individual. This is a feasible strategy when mutations in single cells are rare and the major focus is on individual-level variations. The paper adopts existing methods for bulk eQTL analysis for this task, and comparied the effects of alternative choices at several steps of the pipeline on the final discoveries. It involves a lot of work, but I have some major concerns about the value that can bring to readers.

1. As the paper described, doing eQTL analysis with scRNA-seq data involves many steps, and there are several or many optional choices at each step. This paper did compared the effects of the choices at some steps, but it failed to give any recommendation on the combination of choices at the multiple steps. It didn't give any recommendation on the overall pipeline or strategy, and didn't provide any clue for factors of the data or biological context that need to be considered when making the complicated choices.

2. The criteria used in this paper for comparing the performance of different choices in sc-eQTL studies is the comparison with results of bulk eQTL study. The focus is the number of eGenes in many of the comparisons, and the reproducibility is also considered in some experiments. Having samples with matched scRNA-seq data and bulk RNA-seq data is valuable, but this types of comparison is not convincing enough, and cannot show the advantage of sc-eQTL study. And there is not any biological discussion on what have been discovered and what have been missed with different choices.

3. The descriptions of the simulation model, data and results are rough. It is hard for readers to get the picture of what kind of regulation models were used in the simulation, and therefore it is hard to evaluate whether the recovery of the effect size in the eQTL results is good enough for biological discoveries.

4. The iPSC data are a valuable resource for this study. But as the authors also mentioned, iPSCs are more homogeneous than other types of single cells. It is questionable to what extend the observations in this study can be informative to more complicated single-cell data. The identification of cell types and subtypes is challenging in many tissues.

5. A more fundamental problem of this study is the lack of theoretical analysis on the observations, either from the mathematical aspect or from the biological aspect. There is no analysis or discussion at all on why a certain choice works better on one aspect than another choice. Without such analysis, and considering the challenge in claiming any real data or simulation data can be a good representative for all data, the observations presented in this paper cannot be very helpful for readers to get a deeper understanding on the choices of the methods with regard to their data.

6. There are many places in the paper that only names of methods are given, without any explanation of what they are. Readers cannot get many of the points without looking into the original references. Similarly, many choices in the methods were made without any explanation, except the saying that they are what people use for bulk eQTL studies. Actually, there is not a standard "best practice" in bulk eQTL study either. The authors should discuss the rational underlying the choices. For example, there are other eQTL methods that are not based on LMM. Will the observations change if they are used to replace the LMM method?

7. A paragraph was provided for comparing the strategy of single-cell eQTL with each cell as a sample to the aggregated sc-eQTL study. But the comparison is too brief. Especially, there have been some new methods developed for doing single-cell eQTL analysis. The authors didn't mention them or compare with them.

8. The number of cells that are used in each sample in the aggregation step should be a factor that may influence the results, and this factor should be related with the sequencing platform. The work didn't provide any information on this aspect.

**Reviewer 2**

**Are you able to assess all statistics in the manuscript, including the appropriateness of statistical tests used?**

Yes, and I have assessed the statistics in my report.

**Comments to author:**

This study by Cuomo, Alvari, Azodi et al has examined the statistical approaches available for bulk eQTL analysis in context of the new single cell gene expression measurements. As the authors state, they focus on a statistical modelling approach that is based on current best practices for bulk studies. Their analysis demonstrates that many of these choices are also well-suited for cis eQTL analysis to assess the effect of SNPs on gene mean expression level. The specific strategies evaluated focus on how to aggregate counts across single cells and on covariates to include in the model. The impact of different choices on gene discovery has been demonstrated using two real datasets and simulations. Overall, the choice of statistical models is well-explained and detailed summaries are provided in the main manuscript and its supplements on obtained results. I consider the study a much-needed benchmark for the transition of this field from bulk to single cell studies. The benchmark provided here is of broad interest because it provides an important assessment of best practices on whether adopting well-established bulk methodology is suitable for single cell studies, and towards this end the authors provide several practical guidelines to improve capture of eQTL.

The following questions would be important to clarify:

1) Gene expression level is challenging to estimate in a reliable manner from sparse data (especially the 10X platform) with few cells. In the real datasets used the minimum number of cells per donor is very low (min 5). Why is 5 cells deemed "sufficient" ? Moreover, the cells per donor could vary as much as 1000-fold (7 to 7000 in iPS-neuron data). The authors include a model with sampling variance and demonstrate that considering SV in the model was relevant. However, it is unclear whether this aspect has been addressed in the simulations. Was such low cell number prevalent in real datasets used and did the simulated data match the many-fold differences per simulated "donor"? Clarifying this point would be valuable to also inform experimental design choices upstream of statistical analysis.

2) What type of eQTLs were best captured from both bulk and single cell data? E.g. did this correlate with expression level? Were those variants preferentially located to promoters or distal?

3) The authors refer to iPS as a homogenous cell population. Could they confirm this e.g. by performing unbiased clustering of the integrated sample set and demonstrating that within the obtained clusters (that may capture any sub-structure e.g. driven by cell cycle activity) the proportion of cells per cluster per donor indeed was similar (homogeneous)? It would be important to establish this "homogeneity" from data, since future studies in many cases would start from "isolation" (e.g. clustering-based cell type assignment) of such homogeneous cell populations from more complex tissue samples. The homogeneity achieved could compare against the provided benchmark results.

4) Related to 3: How uniformly were the neuronal cells differentiated across donors or was there biological variability in the degree of differentiation (e.g. position of cells along a pseudotime trajectory)? The authors should also discuss application of their approach in context of developmental studies where the differentiation stage should be accounted for in the analyses. Do the authors have some suggestions how to perform this? Would the strategy be to optimize the clustering (homogeneity)/pseudotime inference or would they expect the PCs to capture such covariates?

5) The authors appear to favour stringent filtering up-front. They discarded 5 runs and also 24 donors. Could the authors demonstrate whether the detection of eGenes would have been dramatically affected had they kept all runs and all donors?

6) The authors settled on validation against the matched bulk sample. With the genotyped sample set, it would be possible to phase the gene regions and use reads spanning exonic het-SNVs to further validate the found eQTLs by quantifying allele-specific expression. Why was this not attempted?

Minor comments:

- The authors should discuss that the effect of SNPs may manifest at other levels of the gene expression distribution, such as variance. For example, variance has been shown to reveal biologically relevant pathways (Grün D. Nat Methods. 2020 doi: 10.1038/s41592-019-0632-3).

- Why would lymphocytes represent a close match to iPS? Other than being experimentally manipulated to become stable cell lines, the actual gene expression profiles would be quite distinct. It appears that metasoft p-vals from GTEX performed well - therefore searching for a "matching" cell type might be less important?

- Do the authors have some estimate on how well they could assign the donor for each cell? In context of their analyses where the minimum number of cells per donor could be relatively small (5 cells) these errors might introduce some variability that the model would not account for.

- The authors refer to quantile normalized data as "gaussianised" ? Would this rather result from both of the consecutive pre-processing steps included : count data log transformation is typically consider to make the value distribution appear more Gaussian. The quantile normalization was performed after this log-transformation. Quantile normalization should also be indicated in schematic fig1.

- The linear mixed model proposed by the authors is well-suited for studies with replicated measurements of the same donor across runs. Do the authors consider this study design optimal for eQTL studies?

- cFDR: could sc-eQTL studies be used instead of bulk?

**Authors Response**

**Point-by-point responses to the reviewers’ comments:**

Reviewer #1 :

Conducting eQTL analysis using scRNA-seq data is a promising direction for studying gene regulation at single-cell resolutions. This paper addresses one possible ways of doing this task: collecting single-cell data of the same cell type and composing pseudo-bulk gene expression data for cells of the same individual. This is a feasible strategy when mutations in single cells are rare and the major focus is on individual-level variations. The paper adopts existing methods for bulk eQTL analysis for this task, and compared the effects of alternative choices at several steps of the pipeline on the final discoveries. It involves a lot of work, but I have some major concerns about the value that can bring to readers.

*We thank the reviewer for their comments and suggestions and we have now addressed their major concerns by adding: i) additional simulation experiments and a more detailed description of the simulation approach, ii) additional replication evidence using GTEx brain eQTL data and allele specific expression, iii) a comparison of different eQTL mapping methodological strategies, and iv) a more in-depth look at the properties of the identified eQTL. We detail our specific responses to all of their comments below.*

1. As the paper described, doing eQTL analysis with scRNA-seq data involves many steps, and there are several or many optional choices at each step. This paper did compared the effects of the choices at some steps, but it failed to give any recommendation on the combination of choices at the multiple steps. It didn't give any recommendation on the overall pipeline or strategy, and didn't provide any clue for factors of the data or biological context that need to be considered when making the complicated choices.

*We thank the reviewer for identifying two important questions a reader may have: 1) what is the recommended overall pipeline or strategy? 2) What factors need to be considered when choosing how to configure a workflow/pipeline for a given study? In fact both of these questions were answered to an extent in the original manuscript and we have attempted to clarify our recommendations for the reader in our revisions.*

*1.In the last section of the results (“Optimised sc-eQTL mapping”) we describe the impact of the combined recommended settings (aggregation/normalization, eQTL mapping method, and multiple testing correction) for sc-eQTL mapping on maximizing power for eQTL discovery in single-cell data. The combined effect of these optimized steps leads to an overall 142% increase in eGene detection compared to the baseline approach. To clarify our recommendations, we have rewritten the last result section to be more explicit about our suggestions, and the impact of these improvements when mapping eQTL in single-cell data (“The previous sections describe how to optimise individual steps during a sc-eQTL mapping workflow, including steps specific to single-cell aggregation and normalization, statistical modelling, and multiple testing correction. Here we describe our combined optimised sc-eQTL mapping workflow and showcase the total increase in power possible when combining the individual findings.”). Thus, the revised manuscript provides clearer recommendations on the best combination of choices at each of the key steps, thereby providing our recommended overall pipeline for sc-eQTL mapping.*

*2. As part of our comparison of aggregation and normalization approaches we test a number of data factors, including sequencing technology (SmartSeq2/10X using both empirical and simulated data), number of donors (using simulations), and now we also include number of cells per donor (using simulations, see reviewer 2 comment #1). Next, as part of our experiment on including sampling variation as a random effect in the model, we also now use simulation results to clarify in what cases including sampling variation is and is not beneficial, a complex, but important consideration. When these factors impact the relative performance of the aggregation methods we highlight this in the text (“We would expect the effect of sampling variation to be the largest when there are large disparities between the number of cells or number of reads between observations, and thus including sampling variation in the model to be most useful in these cases. This is supported by our finding that the addition of the SV random effect resulted in the largest gain and lowest loss in the case of donor-level aggregation on the 10X data.”). Finally, we now include more detailed analyses of the properties of eQTL that are correctly identified by different methods (see response to comment #5), which provides insight into the biological/mathematical context to be considered.*

2. The criteria used in this paper for comparing the performance of different choices in sc-eQTL studies is the comparison with results of bulk eQTL study. The focus is the number of eGenes in many of the comparisons, and the reproducibility is also considered in some experiments. Having samples with matched scRNA-seq data and bulk RNA-seq data is valuable, but this types of comparison is not convincing enough, and cannot show the advantage of sc-eQTL study. And there is not any biological discussion on what have been discovered and what have been missed with different choices.

*We agree that there are limitations to only using the number of eGenes and the replication of eGenes in bulk eQTL mapping to assess performance, and we addressed these limitations by complementing our empirical comparisons and results with extensive simulations. We have also now added additional empirical information as evidence of eQTL replication. Namely, for the 10X floor plate progenitor data we use eQTL mapped from brain tissue by GTEx and, as recommended by reviewer #2 (see comment #6), we use allele-specific expression for both 10X and SmartSeq2 data. Since the simulation results, matched-GTEx, and matched-ASE results tend to support the empirical matched-bulk results, we believe our approach is convincing.*

*We also agree that our study does not show the full power of mapping eQTL in single-cells as opposed to bulk data, however, this is not the aim of the study. The focus of this work is to provide advice on how to optimally perform sc-eQTL mapping, and as such we chose to conduct the assessment of different options to a setting in which as much complexity as possible was controlled - we believe that the iPSC system is ideal for this task as the iPSCs are relatively homogeneous and we have access to perfectly matched bulk and single-cell data, which is all but unique in this field and allows us to answer the technical questions we think are crucially important for the continued progress of the nascent sc-eQTL mapping field. Furthermore, we believe that we, as well as others in the field, have thoroughly demonstrated the advantages of sc-eQTL over bulk-eQTL mapping and believe this is outside of the scope of this work [1,2,3]. We have updated the introduction to highlight more of the established benefits of sc-eQTL mapping (“In addition to deciphering the cellular context in which genetic variants have an effect on gene expression, this single-cell level approach gives us the opportunity to study gene regulation in rare cell types and cell types relevant to a disease or process of interest.” introduction paragraph 2).*

*Finally, the empirical datasets used in this manuscript have already been published, with their original papers describing the biological findings in detail. Given our focus on improving computational workflows for eQTL mapping in single-cell data, we have added more information on the statistical characteristics of eGenes and eSNPs that were discovered or missed with different method choices (Please see our response to comment # 5 for details).*

*[1]	Cuomo et al: https://www.nature.com/articles/s41467-020-14457-z*

*[2]	Jerber et al: https://www.nature.com/articles/s41588-021-00801-6*

*[3]	van der Wijst et al: https://www.nature.com/articles/s41588-018-0089-9*

3.	The descriptions of the simulation model, data and results are rough. It is hard for readers to get the picture of what kind of regulation models were used in the simulation, and therefore it is hard to evaluate whether the recovery of the effect size in the eQTL results is good enough for biological discoveries.

*We have now extended the description of the simulations in the methods of the manuscript, specifically including more information about the model used to simulate scRNA-seq data and how the eQTL effects are incorporated into those simulations. We also provide the code used to generate our simulations with documentation explaining the necessary steps and parameters used (https://github.com/single-cell-genetics/optimising_singlecell_eqtl_paper). Finally, we provide a reference to a vignette describing splatPop in detail (http://www.bioconductor.org/packages/devel/bioc/vignettes/splatter/inst/doc/splatPop.html).*

4.	The iPSC data are a valuable resource for this study. But as the authors also mentioned, iPSCs are more homogeneous than other types of single cells. It is questionable to what extend the observations in this study can be informative to more complicated single-cell data. The identification of cell types and subtypes is challenging in many tissues.

*We agree that cell type identification is often a challenging problem and poor cell type annotation may negatively impact sc-eQTL mapping. However, the scope of this paper is to cover the eQTL mapping workflow *after* the identification/clustering of cells into cell types or subtypes. The reason for this is that cell (sub) type annotation is often biological system/dataset/technology specific and has already been thoroughly benchmarked [1], which we now highlight in the introduction (“These processing steps are critical for successful downstream analyses like sc-eQTL mapping. However, because these processing steps are largely dataset- and/or technology-dependent and have been previously reviewed and benchmarked, here we focus on the steps after processing that are specific to eQTL mapping.” introduction paragraph 4).*

*Since a sensible and common approach for eQTL discovery in heterogeneous/ complicated single-cell data is to analyse each cell (sub) type separately, we see any homogeneous population of cells (such as the iPSCs) as a good model for such an analysis. However, we also included a more heterogeneous single-cell dataset: cells annotated as floor plate progenitor cells in a population of cells differentiating from iPSCs toward dopaminergic neurons sequenced using 10X technology (see results section: 10X datasets). We have added language to that section to highlight that the 10X data is an example of a more complex cell population (“We used data from a recent differentiation study of iPSCs toward dopaminergic neurons*

*[13]. This dataset was more complex than the iPSC Smart-Seq2 dataset described above, as it contained cells from multiple cell types. To map cell-type specific sc-eQTL, we selected only cells annotated as midbrain floor plate progenitor (FPP) cells (n=174, Methods).”) and added to our discussion an acknowledgement that poor pre-processing, including cell type assignment, could result in poor eQTL mapping (“4) single-cell processing steps such as expression quantification, cell-level QC, and cell-type annotation could effect eQTL mapping performance, but best practices for these steps are likely to be dataset and technology specific rather than specific for sc-eQTL mapping, and were thus not assessed here.”).*

*[1] Cortel et al: https://www.nature.com/articles/s41587-021-00896-6*

5. A more fundamental problem of this study is the lack of theoretical analysis on the observations, either from the mathematical aspect or from the biological aspect. There is no analysis or discussion at all on why a certain choice works better on one aspect than another choice. Without such analysis, and considering the challenge in claiming any real data or simulation data can be a good representative for all data, the observations presented in this paper cannot be very helpful for readers to get a deeper understanding on the choices of the methods with regard to their data.

*We thank the reviewer for their suggestion to provide insights into the mathematical aspects driving differences in eQTL discovery with the different approaches. For our simulation data, we compared eGenes that were correctly identified using different aggregation approaches in terms of: simulated gene mean, gene variance, and eQTL effect size. Similarly for the empirical data we looked at the discovery of bulk eQTL in the single cell eQTL results, here we additionally looked into the distance between the lead variant and the eQTL gene.*

*Findings from this analysis were added to the manuscript (empirical results Fig. S3 “Unsurprisingly, we find that strong eQTL effects can be picked up using all methods, and eGenes with high expression and low variance are also easier to be identified.” and simulated results Fig. S6 “Further, by comparing the mathematical properties of the eQTL correctly detected by each aggregation method, we can tell that median aggregation tended to miss eQTL for genes with high variance and low mean expression, while sum aggregation tended to miss eQTL with small effect sizes.”). In summary, compared to eGenes that were found by all three aggregation approaches: 1) median aggregation tends to miss eGenes with high variance and low gene means, 2) sum aggregation tends to miss eQTL with smaller effect sizes, and 3) mean aggregation missed few eGenes that the other methods were able to capture. Results from the simulations also highlight that the eQTL missed by all aggregation methods tended to have smaller effect sizes, lower expression, and higher variance. Finally, results from the empirical data detect no effect of the distance between the eSNP and eGene on single-cell replication of bulk eQTL in iPSCs (Fig. S3 and S6).*

*Figures S3 (empirical) and S6 Simulations)*

*Additionally, we considered eQTL identified in single-cell data (using the ‘dr-mean’ aggregation strategy), and compared between sc-eQTL that 1) could be replicated in the bulk results (both m-bulk and a-bulk), 2) could only be replicated in bulk results using all samples (a-bulk) but not when limiting to the same donors considered for single-cell data (m-bulk), 3) could not be replicated in bulk (neither m-bulk nor a-bulk). (No sc-eQTL could be replicated in m-bulk but not in a-bulk.) Again, we compared eQTL discovery in terms of gene mean, variance and eQTL effect size, both in the bulk data and in the single-cell data (Figure S4). From this analysis, we find that sc-eQTL that cannot be replicated in bulk tend to have a smaller effect size both in the single-cell and, even more evidently, in the bulk results. We have added these results in the main text (“When investigating which single-cell eQTL could be replicated in the bulk results, we noted that this was mostly linked to the eQTL effect size, both in the single-cell and bulk eQTL results (Fig. S4)”), and have added the figure below (Fig. S4) to the supplementary information.*

*6. There are many places in the paper that only names of methods are given, without any*

*explanation of what they are. (A) Readers cannot get many of the points without looking into*

*the original references.*

*We thank the reviewer for pointing this out. We have ensured that all methods referred to in the paper now include short descriptions in the Methods section. Specifically, we have now added a brief description of the methods used to capture global expression covariates, i.e. PEER, MOFA and scVAE (“first, we considered Probabilistic Estimation of Expression Residuals (PEER [38], a method designed specifically to account for global expression confounders in the context of (bulk) eQTL mapping; next, we considered Multi-Omics Factor Analysis (MOFA, which we used in two flavours: with and without sparsity; [38], [40]). While originally designed for multi-omic data, MOFA can be applied when considering a single omic layer (gene expression in this case) to probabilistically identify common sources of variation across all genes, and has been applied to a variety of bulk and single-cell datasets. Finally, we tested for this purpose a method called Linearly Decoded Variational AutoEncoder (LDVAE [39], [40]. This model also aims at finding factors that co-vary across all features (i.e. genes) considered, and is especially designed for single-cell data.”).*

(B) Similarly, many choices in the methods were made without any explanation, except the saying that they are what people use for bulk eQTL studies. Actually, there is not a standard "best practice" in bulk eQTL study either. The authors should discuss the rational underlying the choices. For example, there are other eQTL methods that are not based on LMM. Will the observations change if they are used to replace the LMM method?

*Our reason for selecting linear mixed models (LMMs) is that LMMs are the only model for eQTL mapping that can properly account for cryptic relatedness and population structure, thus allowing us to deal with replicated genetic donors. However, we agree with the reviewer that even for bulk eQTL mapping, many different statistical methods are used, with no clear “best practice”. To address this, we have added a comparison of commonly used eQTL mapping strategies including correlation-based eQTL mappings (pearson and spearman), a linear model (LM), and using a linear mixed model (LMM) for eQTL mapping (Table S1, Fig. S1). We find that there is a very limited difference in discovery power of eQTL between the methods (89-92% of the eGenes are found in all methods), in other words, most eQTL are found regardless of method. We do however see that replication is slightly better (1-2%) when using a LMM based model as compared to the correlation based methods. The model comparison we present here in the context of single-cell eQTL mapping in principle also holds for bulk eQTL mapping.*

*Table S1*

*Figure S1*

7. A paragraph was provided for comparing the strategy of single-cell eQTL with each cell as a sample to the aggregated sc-eQTL study. But the comparison is too brief. Especially, there have been some new methods developed for doing single-cell eQTL analysis. The authors didn't mention them or compare with them.

*We have adjusted the structure of our manuscript to have a dedicated section for our results on single-cell eQTL mapping without aggregation (see “Mapping eQTL using individual cell expression profiles” (page 13, line: 332). In this section we expand our discussion of our original results and include the additional experiments described below.*

*As mentioned by the reviewer, recently there have been a few single-cell eQTL mapping methods proposed that do not require aggregation as explored here. However, most of these papers are doing sc-eQTL mapping in a different setting to what we are presenting. For instance, BaseQTL [1], is designed to work on single cell expression data by itself, i.e. it doesn’t require DNA genotyping data. These methods come with the drawback that only variants that are covered in the single-cell RNA seq reads can be investigated.*

*The only new method we are aware of that is applicable to the setting we study, is SCeQTL [2], which uses zero-inflated negative binomial regression to perform single-cell eQTL mapping. We originally did not include this method because it is computationally very expensive compared to our standard linear (mixed) models, and because it cannot account for repeated measures. However, we have now included results using SCeQTL to map eQTL in one of our simulated datasets. Because SCeQTL is a computationally intensive approach, we were only able to apply it to a subset of the data (10 cells per donor = 870 cells total). Compared to using our LMM on the same subset of cells, SCeQTL had similar eQTL detection power, but made even more false discoveries, and took ~11x longer to run (Figure S9).*

*Figure S9*

*Given the required runtime and high false discovery rates from both running LIMIX and especially SCeQTL when running on individual single cells, and large numbers of tests involved in eQL mapping, we feel that without further method optimization the aggregation methods proposed are a very sensible compromise.*

*[1]	BaseQTL: https://www.biorxiv.org/content/10.1101/2020.07.16.203851v1.full-text*

*[2]	SCeQTL : https://bmcbioinformatics.biomedcentral.com/articles/10.1186/s12859-020-3534-6*

8. The number of cells that are used in each sample in the aggregation step should be a factor that may influence the results, and this factor should be related with the sequencing platform. The work didn't provide any information on this aspect.

*We have included a new analysis to study how changes in the number of cells available per donor impacts sc-eQTL mapping using our simulation framework. As expected, power improves when counts from more cells are aggregated (see Figures S7B for SmartSeq2 and S11B for 10X). In the empirical data, we simply used all cells available for each sample, and we now report such statistics in Figure S2C-D.*

*Figures S7b (smartseq2) and S11b (10X)*

*As mentioned by the reviewer, the number of cells and therefore number of reads per donor / donor-run does influence the power of mapping eQTL, which is the reason why we explore the approach accounting for sampling variation. To get a better understanding of these effects we have also extended the simulation results on this part of the manuscript (see response to reviewer #2, comment #1).*

Reviewer #2

This study by Cuomo, Alvari, Azodi et al has examined the statistical approaches available for bulk eQTL analysis in context of the new single cell gene expression measurements. As the authors state, they focus on a statistical modelling approach that is based on current best practices for bulk studies. Their analysis demonstrates that many of these choices are also well-suited for cis eQTL analysis to assess the effect of SNPs on gene mean

expression level. The specific strategies evaluated focus on how to aggregate counts across single cells and on covariates to include in the model. The impact of different choices on gene discovery has been demonstrated using two real datasets and simulations. Overall, the choice of statistical models is well-explained and detailed summaries are provided in the main manuscript and its supplements on obtained results. I consider the study a much-needed benchmark for the transition of this field from bulk to single cell studies. The benchmark provided here is of broad interest because it provides an important assessment of best practices on whether adopting well-established bulk methodology is suitable for single cell studies, and towards this end the authors provide several practical guidelines to improve capture of eQTL.

*We thank the reviewer for their close reading of our manuscript and their appreciation for the importance and broad interest of the results we present, as well as for their comments and suggestions.*

*As mentioned above, we have now made substantial revisions to our manuscript following their and Reviewer 1’s comments. Specifically, we have now addressed their concerns by adding: i) additional simulation experiments and a more detailed description of the simulation approach, ii) additional replication evidence using GTEx brain eQTL data and allele specific expression, iii) a comparison of different eQTL mapping methodological strategies, and iv) a more in-depth look at the properties of the identified eQTL.*

The following questions would be important to clarify:

1) Gene expression level is challenging to estimate in a reliable manner from sparse data (especially the 10X platform) with few cells. In the real datasets used the minimum number of cells per donor is very low (min 5). Why is 5 cells deemed "sufficient" ? Moreover, the cells per donor could vary as much as 1000-fold (7 to 7000 in iPS-neuron data). The authors include a model with sampling variance and demonstrate that considering SV in the model was relevant. However, it is unclear whether this aspect has been addressed in the simulations. Was such low cell number prevalent in real datasets used and did the simulated data match the many-fold differences per simulated "donor"? Clarifying this point would be valuable to also inform experimental design choices upstream of statistical analysis.

*We thank the reviewer for these helpful suggestions. To clarify, the simulations were matched to the characteristics from the actual samples. In particular, the number of cells simulated for each donor was sampled from a gamma distribution estimated from the number of cells/donor from the empirical data, thus the simulations do match the many fold differences in cells/donor seen in the empirical data. We have clarified this point in the methods section (“The number of cells to simulate per donor was sampled from a gamma distribution estimated from the number of cells per donor from the empirical data.”). We have also now included an analysis of the impact of considering sampling variation (SV) as a random effect for sc-eQTL mapping on our simulations. This analysis supports our finding from the empirical data that SV as a random effect can improve power to identify sc-eQTL, however from the simulations we also observe an increased false positive rate (Table S11). Important to note here is that the effect of the sampling variation is going to be largest with large disparities between the number of cells or reads between the observations, which is not completely reflected in the simulations.*

*Table S11*

*We have also included the number of cells-per-donor as an experimental variable in our study (see response to reviewer #1, comment #8).*

*Finally, regarding using 5 cells as a minimum threshold for inclusion in the model, this threshold was selected to balance the need to remove donors with lower quality counts (due to having few cells sequenced) with the need to include as many donors as possible to maximize eQTL mapping power. For the empirical SmartSeq2 data, only one donor had fewer than 20 cells, so even a 4x increase in the minimum threshold would have had little impact on our results. However, we agree with the reviewer that this threshold could be important, especially for studies with fewer cells sequenced. We have added a discussion of this issue in our (Figure S2c,d) (“Note that all donors from the iPSC Smart-Seq2 dataset had > 5 cells per donor, so this threshold only applied when aggregating over donor-run. In the 10X data, only five donors were filtered out by this filter (Fig. S2c,d). In cases with low sequencing coverage or when poor sequencing quality is a concern, a higher minimum cell threshold may be needed, with the trade-off being fewer donors and thus less power for eQTL discovery [18].”).*

2) What type of eQTLs were best captured from both bulk and single cell data? E.g. did this correlate with expression level? Were those variants preferentially located to promoters or distal?

*We thank the reviewer for this excellent question. We have added a more detailed comparison of what drives the discovery power of induced effects in the simulations (Fig. S6), and similarly treated the bulk effects as ground truth and assessed which effects we could also discover in the empirical data (Figure S3) (see response to reviewer #1 comment # 5 for figures and key observations).*

3) The authors refer to iPS as a homogenous cell population. Could they confirm this e.g. by performing unbiased clustering of the integrated sample set and demonstrating that within the obtained clusters (that may capture any sub-structure e.g. driven by cell cycle activity) the proportion of cells per cluster per donor indeed was similar (homogeneous)? It would be important to establish this "homogeneity" from data, since future studies in many cases would start from "isolation" (e.g. clustering-based cell type assignment) of such homogeneous cell populations from more complex tissue samples. The homogeneity achieved could compare against the provided benchmark results.

*The homogeneity of gene expression in iPS cells is described in multiple other papers, specifically both Kilpinen et al [1] and Bonder et al [2] have shown this on samples from the HipSci study. Furthermore, the homogeneity of these specific single cells are assessed in the Cuomo et al [3] study, where the full single cell dataset was explored and little difference was observed between the iPS cells.*

*As the reviewer also outlines, for the FPP cells (10X data) we do use a clustering-based cell type assignment. We see that the downstream optimizations have very similar effects in exactly this setting. More information on the cell type identification can be found in the original publication on the dataset (Jerber et al [4]).*

*[1]	Kilpinen et al: https://www.nature.com/articles/nature22403*

*[2]	Bonder et al: https://www.nature.com/articles/s41588-021-00800-7*

*[3]	Cuomo et al: https://www.nature.com/articles/s41467-020-14457-z*

*[4]	Jerber et al: https://www.nature.com/articles/s41588-021-00801-6*

4) Related to 3: How uniformly were the neuronal cells differentiated across donors or was there biological variability in the degree of differentiation (e.g. position of cells along a pseudotime trajectory)? The authors should also discuss application of their approach in context of developmental studies where the differentiation stage should be accounted for in the analyses. Do the authors have some suggestions how to perform this? Would the strategy be to optimize the clustering (homogeneity)/pseudotime inference or would they expect the PCs to capture such covariates?

*We thank the reviewer for raising these important questions; we will unpack those questions below.*

*First, as discussed in the response to Reviewer 1’s Comment #4, cell type identification is often a challenging problem and poor cell type annotation may negatively impact sc-eQTL mapping. However, the scope of this paper is to cover the eQTL mapping workflow *after* the identification/clustering of cells into cell types or subtypes. The reason for this is that cell (sub) type annotation is often dataset/technology specific and has already been thoroughly benchmarked [1]; our recommendations are agnostic to the exact approach taken to cell type identification and annotation.*

*Since a sensible and common approach for eQTL discovery in heterogeneous/complex single-cell data is to analyse each cell (sub) type separately, we see any homogeneous population of cells (such as the iPSCs) as a good model for such an analysis. Additionally, we included the 10X FPP cells to represent a cell type that was annotated from a more heterogeneous single-cell dataset. We have added language to that section to highlight that the 10X data is an example of a more complex cell population (“We used data from a recent differentiation study of iPSCs toward dopaminergic neurons [13]. This dataset was more complex than the iPSC Smart-Seq2 dataset described above, as it contained cells from multiple cell types. To map cell-type specific sc-eQTL, we selected only cells annotated as midbrain floor plate progenitor (FPP) cells (n=174, Methods).”) and added to our discussion an acknowledgement that poor processing, including cell type assignment, could result in poor eQTL mapping (“4) single-cell processing steps such as expression quantification, cell-level QC, and cell-type annotation could effect eQTL mapping performance, but best practices for these steps are likely to be dataset and technology specific rather than specific for sc-eQTL mapping, and were thus not assessed here.”).*

*The cell type annotation for the 10X FPP cells is taken from the original study [2], where more information can be found on the specific methods for clustering and cell type annotation. Given the long intervals between the single-cell expression profiling experiments in the study (time points used are day11, day30 and day52 of neuronal differentiation), cells were not placed on a continuous pseudotime trajectory and differences in differentiation efficiency are only assessed at later time points of the differentiation study (i.e. day52). Any variation that is already present at day 11, the time point of the FPP cells we used, is expected to be corrected for using expression PCA in the eQTL model. In the Cuomo et al differentiation eQTL paper [3] a more fine grained time series is available for the shorter endoderm differentiation (day0-3). Here the authors used known differentiation stage gene markers combined with pseudotime to refine cell type annotation, and show that this increases eQTL mapping power [3]. Additionally, they used interaction eQTL and allele specific expression-based interaction mapping methods to explore dynamics of genetic effects. These analyses are downstream of the initial eQTL mapping, as most of them used lead eQTL as starting points. Therefore, and because this is so far the only paper exploring this space (using single-cell data), we feel that it is too early to do a benchmark on that specific analysis and it falls largely out of scope of our current message.*

*[1]	Cortel et al: https://www.nature.com/articles/s41587-021-00896-6*

*[2]	Jerber et al: https://www.nature.com/articles/s41588-021-00801-6*

*[3]	Cuomo et al: https://www.nature.com/articles/s41467-020-14457-z*

5)	The authors appear to favour stringent filtering up-front. They discarded 5 runs and also 24 donors. Could the authors demonstrate whether the detection of eGenes would have been dramatically affected had they kept all runs and all donors?

*To get an insight into this we redid the normalisation and aggregation steps this time considering all cells, without performing any cell QC. By not excluding the five runs, we added 9 extra donors and a total of 26 donor-run combinations. Some of the numbers mentioned by the reviewer are confounded by either overlapping donors between runs, due to non-european ancestry, or being from a monogenic diabetes line (not for cell-level QC). When mapping sc-eQTL considering these additional samples we observe 146 extra eQTL effects (0.7%) in the dr-mean setting (see table below). However, we also observe a strong decrease in replication of eQTL (-9%) in bulk indicating that the QC has removed low quality single cells and therefore the eQTL	are more robust	after QC:*

*Furthermore, we overlapped our non-standard filtering approach with a more traditional single cell QC filtering, i.e. filtering for number of expressed genes, and observed high concordance (Figure S13).*

6)	The authors settled on validation against the matched bulk sample. With the genotyped sample set, it would be possible to phase the gene regions and use reads spanning exonic het-SNVs to further validate the found eQTLs by quantifying allele-specific expression. Why was this not attempted?

*We thank the reviewer for this useful suggestion. We have now included replication of eQTL using allele-specific expression (ASE) for all analyses using the iPSC data (Table S4 and Table S7). For ASE we used WASP [1] and merged all cells from a donor into one sample file before ASE testing. Information on the input for WASP is added to the methods (Methods section: “Allele specific expression quantification and testing” (Page: 24, line: 679)). From this analysis, we found that the replication of single cell eQTL in single cell ASE (-35%) is much lower than the replication of single cell eQTL in bulk expression data (-70%). Possible reasons for why fewer effects could be replicated using ASE include: i) the use of a filtered gene annotation file that only contains unique non-overlapping regions of genes (as required for WASP), ii) the much lower read counts as compared to bulk expression and read filtering before ASE counting, iii) in some cases poor phasing of variants and finally iv) because of the different test used for ASE versus eQTL mapping. Indeed, when only considering effect direction in the replicable ASE effects replication is much higher (-75%).*

*As further validation of our observations, we also added replication of the eQTL effects on the 10X data. Given that there is no matching bulk reference for the neuronal cell type considered in the 10X data, we assessed replication across the brain tissues available in GTEx v7 [3]. The results are added in the relevant 10X supplementary tables (Table S6).*

*[1]	van de Geijn et al: https://www.nature.com/articles/nmeth.3582*

*[2]	Bonder et al: https://www.nature.com/articles/s41588-021-00800-7*

*[3]	GTEx et al: https://www.nature.com/articles/nature24277*

*Minor comments:*

- The authors should discuss that the effect of SNPs may manifest at other levels of the gene expression distribution, such as variance. For example, variance has been shown to reveal biologically relevant pathways (Grün D. Nat Methods. 2020 doi: 10.1038/s41592-019-0632-3).

*The study of the genetic control of expression variability is an interesting but complicated issue. This is partly due to the difficulty to disentangle the mean and the variance of count data. Moreover, these effects have been shown to be much weaker than mean-level ones. For example Sarkar et al. (Plos Genetics, 2019 doi: 10.1371/journal.pgen.1008045) have shown that a minimum sample size of 4,015 individuals would be necessary to achieve 80% power to detect the strongest dispersion QTL. Moreover, the majority of effects on variance have been shown to be trans effects (Morgan et al., Plos Genetics, 2020 doi: 10.1371/journal.pgen.1008686), which require much larger sample size than we have here. Given these considerations, we focused on expression QTL only, and leave the study of variance eQTL using single-cell data for future work.*

- Why would lymphocytes represent a close match to iPS? Other than being experimentally manipulated to become stable cell lines, the actual gene expression profiles would be quite distinct. It appears that metasoft p-vals from GTEX performed well - therefore searching for a "matching" cell type might be less important?

*We thank the reviewer for raising this question and due to the possibility of misunderstandings have rephrased this section in the text. The matching between the tissues of GTEx and iPSC is detailed in Bonder et al [1], but indeed using the metasoft p-values gave a comparable result. It is important to note here that a tissue with a gene expression profile that is as similar as possible to the target cell type in the sc-eQTL mapping is important. In the GTEx metasoft meta-analysis only genes expressed in all tissues are meta-analyzed, i.e. almost all genes that are tissue specific are dropped, hindering the re-use of these metasoft eQTL statistics in this specific setting, this means that a single tissue or meta analysis statistics without filtering genes are still the better way to go. The relevant text sections now read: “To generalise this approach we applied cFDR using other reference eQTL statistics to guide our sc-eQTL mapping analysis. Specifically, we selected two external reference sets from GTEx: 1) the lymphocyte eQTL summary statistics from GTEx (v7), and 2) the meta-tissue eQTL results by GTEx (v7).” and “Of importance here is that for Table S13 we subsetted down to only consider genes tested in all datasets, to be able to compare between cFDR conditionings. When assessing the impact per reference p-value set, we see that leveraging the joint cell types from the Cuomo et al. study yields a comparable number of discoveries as using the m-bulk data (2,821 vs 2,887 eGenes, with comparable replication fractions), and observe that the GTEx summary statistics do considerably worse as the number of overlapping expressed genes is much lower (Table S14).”*

*[1] Bonder et al: https://www.nature.com/articles/s41588-021-00800-7*

- Do the authors have some estimate on how well they could assign the donor for each cell? In context of their analyses where the minimum number of cells per donor could be relatively small (5 cells) these errors might introduce some variability that the model would not account for.

*We are very confident in our donor assignment procedure. The model used for the iPSC data (i.e. Cardelino [1]) performed extremely well, with 99% of QC-passing cells being confidently assigned to a donor of origin. In the 10X study demuxlet [2] is used, which also has shown to perform very well (>95% detection with more than 50 variants deteced) [1,2]. Both these methods are shown to be very accurate especially in the setting where donor genotypes are available and in our specific settings we do know the lines included in a specific pool, so we verified that the donor a cell is assigned to is indeed in that pool. More information on the donor identification can be found in the original publications [3,4].*

*[1]	McCarthy et al: https://www.nature.com/articles/s41592-020-0766-3*

*[2]	Kang et al: https://www.nature.com/articles/nbt.4042*

*[3]	Jerber et al: https://www.nature.com/articles/s41588-021-00801-6*

*[4]	Cuomo et al: https://www.nature.com/articles/s41467-020-14457-z*

- The linear mixed model proposed by the authors is well-suited for studies with replicated measurements of the same donor across runs. Do the authors consider this study design optimal for eQTL studies?

*We thank the reviewer for this comment, and refer to the response to reviewer 1, comment 6. Briefly, indeed, we regard LMMs as optimally suited for genetic analyses in general, and particularly in the presence of underlying structure across the sample considered, including in the case of replicated measurements. To illustrate this point we have now included a comparison with alternative common methods including a linear regression and correlation-based strategies (Figure S1, Table S1).*

- cFDR: could sc-eQTL studies be used instead of bulk?

*In our case, the idea was to leverage studies with more power, which are currently mostly bulk, but we also present the use of single cell expression data for the cFDR procedure (“Lastly, we used a cell-type agnostic eQTL mapping on the two other differentiation time points presented in the Cuomo et al study (i.e. mesendoderm and definitive endoderm). This final setting should be useful in most common sc-eQTL mapping settings where either a cell-type specific or cell-type agnostic analysis can be performed within the same study.”)*

- The authors refer to quantile normalized data as "gaussianised" ? Would this rather result from both of the consecutive pre-processing steps included : count data log transformation is typically consider to make the value distribution appear more Gaussian. The quantile normalization was performed after this log-transformation. Quantile normalization should also be indicated in schematic fig1.

*We thank the reviewer for raising this point, indeed we use the term “gaussianised” to reflect a quantile normalization towards a gaussian distribution. The quantile normalization step has now been added to the schematic Fig. 1 (panel c), and we clarify this technical step in Methods (“The expression profile of each gene was quantile normalised to a standard normal distribution”).*

*Fig 1*

**Second round of review**

**Reviewer 2**

The authors have made several improvements to the manuscript and there are no major concerns remaining.

Minor comments:

line 202: "Overall, we observe two main trends." Re-phrase the sentence to be more specific. The paragraph focuses on the number of discovered eGenes. It would be helpful to guide the reader to focus on the data presented in the first column of Table 1 (it becomes unambiguous only at the second comparison, where the specific values discussed can be checked against those in the table).

Supplementary tables: Check the correspondence of Table numbering and Results text. E.g. the ASE data and all HVG results are presented in Table S4 (in text S5).

Methods: Include version information for tool packages used in Methods text, as many methods are under development with regular updates. There is also lack of detail in the description of the method-specific parameters used for the normalization test, which could be addressed at the code repository level. Currently, the readme in the main code repository is not referencing the additional files in the separate repository that perhaps include this. It was not possible to check this, as the code repository https://github.com/single-cell931genetics/optimising_singlecell_eqtl_paper is not accessible. Make sure both repositories are set to public status.

**Authors Response**

**Point-by-point responses to the reviewers’ comments:**

Comments by reviewer 2:

Minor comments:

line 202: "Overall, we observe two main trends." Re-phrase the sentence to be more specific. The paragraph focuses on the number of discovered eGenes. It would be helpful to guide the reader to focus on the data presented in the first column of Table 1 (it becomes unambiguous only at the second comparison, where the specific values discussed can be checked against those in the table).

*RE: We thank the reviewer for this suggestion. We have now included the relevant numbers for the comparison also in the main text.*

Supplementary tables: Check the correspondence of Table numbering and Results text. E.g. the ASE data and all HVG results are presented in Table S4 (in text S5).

*RE: We thank the reviewer for their careful check of the manuscript and apologize for this referencing error.*

A) Methods: Include version information for tool packages used in Methods text, as many methods are under development with regular updates.

*RE: We thank the reviewer for pointing out this issue, we have now included versions of tools used in the methods section.*

B) There is also lack of detail in the description of the method-specific parameters used for the normalization test, which could be addressed at the code repository level. Currently, the readme in the main code repository is not referencing the additional files in the separate repository that perhaps include this. It was not possible to check this, as the code repository https://github.com/single-cell931genetics/optimising_singlecell_eqtl_paper is not accessible. Make sure both repositories are set to public status.

*RE:Probably as a consequence of the double page numbering the link was not properly copied. The correct link: https://github.com/single-cell-genetics/optimising_singlecell_eqtl_paper is available and correctly linked in the manuscript.*
